# CRISPR/Cas9-Directed PEPD Targeting Is Associated with Reduced PEPD Activity and an Exploratory p53- and Apoptosis-Related Protein Pattern in MCF7 Breast Cancer Cells

**DOI:** 10.3390/molecules31193360

**Published:** 2026-09-22

**Authors:** Magda Chalecka, Dominika Stencel, Jerzy Palka, Arkadiusz Surazynski

**Affiliations:** 1 Department of Medicinal Chemistry, Faculty of Pharmacy, Medical University of Bialystok, Mickiewicza 2D, 15-222 Bialystok, Poland; magda.chalecka@umb.edu.pl (M.C.); jerzy.palka@umb.edu.pl (J.P.); 2 Department of Pharmaceutical Chemistry, Faculty of Pharmaceutical Sciences in Sosnowiec, Medical University of Silesia in Katowice, Jagiellońska 4, 41-200 Sosnowiec, Poland; dominika.stencel@sum.edu.pl

**Keywords:** prolidase, PEPD, CRISPR/Cas9, MCF7, p53, MDM2, apoptosis

## Abstract

Prolidase (PEPD) is a metalloproteinase that hydrolyzes imidodipeptides containing proline or hydroxyproline at the C-terminus and also participates in the non-catalytic regulation of cellular stress responses and p53 availability. Here, we characterized population-level responses associated with transient CRISPR/Cas9-directed targeting of PEPD in heterogeneous MCF7 breast cancer cultures using three independent sgRNAs. PEPD enzymatic activity was quantified in three independent biological experiments, whereas PEPD and p53/apoptosis-related proteins were examined by representative Western blotting and exploratory immunofluorescence microscopy. All three PEPD-targeting constructs were associated with reduced population-average PEPD activity, and representative immunoblots showed directionally concordant reductions in PEPD protein signal. Representative protein analyses additionally revealed a qualitative pattern involving p53, MDM2, cleaved caspase-9, cleaved caspase-7, and cleaved PARP-1. These protein observations were not supported by independent biological replication suitable for inferential analysis and are therefore interpreted descriptively. Editing of the endogenous PEPD locus was not directly confirmed or quantified by genomic analysis. During prolonged puromycin selection, the persistence of adherent PEPD-targeted cells decreased under the conditions tested; however, this experiment was qualitative and was not designed as a formal viability or rescue assay. Collectively, the data demonstrate a replicated population-level reduction in PEPD enzymatic activity following PEPD-directed CRISPR/Cas9 perturbation and identify an associated exploratory p53/MDM2- and apoptosis-related protein pattern. A contribution of Cas9-mediated DNA-damage signaling to the latter cannot be excluded.

## 1. Introduction

Prolidase (peptidase D; PEPD, EC 3.4.13.9) is a cytoplasmic exopeptidase that catalyzes the hydrolysis of imidodipeptides and imidotripeptides containing proline or hydroxyproline at the C-terminal position [1]. As an enzyme involved in the final step of collagen-derived iminodipeptide degradation, PEPD plays a crucial role in intracellular proline recycling, extracellular matrix remodeling, and collagen resynthesis [2,3]. Due to the high abundance of proline and hydroxyproline in extracellular matrix proteins, PEPD activity is essential for maintaining tissue homeostasis, wound healing, and cellular metabolism [4].

In humans, the PEPD gene is located on chromosome 19 and consists of 15 exons. Its product is a 493-amino-acid polypeptide with a molecular weight of 54 kDa [5,6]. The active form of human PEPD exists as a symmetrical homodimer, in which each subunit contains a small N-terminal domain and a larger catalytic domain belonging to the metalloprotease family. Manganese is essential for the activity of human PEPD [7,8,9]. PEPD has also been shown to undergo phosphorylation on tyrosine residues contributing to an increase in the enzyme activity [10,11]. However, the importance of PEPD in cellular metabolism is illustrated in conditions of PEPD deficiency. This is a rare autosomal recessive disorder caused by mutations in the PEPD gene, and is characterized by reduced PEPD activity, iminodipeptiduria, and a broad spectrum of clinical manifestations, including chronic leg ulcers, impaired wound healing, recurrent infections, splenomegaly, developmental delay, dysmorphic features, and autoimmune symptoms resembling systemic lupus erythematosus [12,13,14,15,16]. In severe cases, the disease may result in premature death [17,18]. The multisystemic phenotype of PEPD deficiency indicates that loss of PEPD affects several aspects of tissue and cellular homeostasis; however, the relative contribution of impaired catalytic activity and non-catalytic PEPD functions remains incompletely defined.

Over the last decades, increasing evidence has highlighted the importance of PEPD in cancer biology. Numerous studies have reported that PEPD expression and activity may increase tumor aggressiveness, and elevated PEPD activity correlates with tumor progression, extracellular matrix remodeling, invasiveness, and the ability of cancer cells to adapt to unfavorable microenvironmental conditions [19]. The role of PEPD in these processes is partly related to its catalytic activity, since the products of PEPD-dependent hydrolysis, particularly proline, can be incorporated into metabolic pathways associated with protein biosynthesis, the maintenance of redox homeostasis, mitochondrial function, and energy production [4,20].

Under tumor-associated stress conditions, including hypoxia, oxidative stress, and nutrient deprivation, proline metabolism may play an important role in cellular adaptation [21,22]. Proline participates in the regulation of intracellular redox balance and energy metabolism through pathways associated with the proline dehydrogenase/proline oxidase, pyrroline-5-carboxylate, pyrroline-5-carboxylate reductase (PRODH/POX–P5C–PYCR) axis [23]. Within the proline cycle, proline is converted into P5C by PRODH/POX, a process accompanied by ATP generation and reactive oxygen species (ROS) production [24,25,26]. Depending on the metabolic context, this pathway may activate apoptosis or autophagy [20,27]. Since the proline cycle is interconnected with the tricarboxylic acid cycle, the urea cycle, and the pentose phosphate pathway, it plays an important role in the regulation of cellular energy status, nucleotide biosynthesis, and biomass production, thereby influencing cell survival, growth, and proliferation [20]. Therefore, PEPD activity may facilitate the metabolic adaptation of cancer cells by providing substrates that support survival under conditions of limited oxygen and nutrient availability [28].

PEPD activity and the resulting availability of proline may also promote the stabilization of hypoxia-inducible factor 1α (HIF-1α) and enhance the expression of HIF-1α target genes, including glucose transporter 1 (GLUT1) and vascular endothelial growth factor (VEGF) [29]. Therefore, an increase in the angiogenic potential of cancer cells is accompanied by a metabolic shift toward glycolysis, thereby supporting ATP production under hypoxic conditions [4,29]. Extracellular PEPD can also interact with members of the ErbB receptor family, although its effects on EGFR and HER2/ErbB2 are not equivalent. PEPD directly binds and activates EGFR independently of its enzymatic activity, thereby stimulating downstream signaling. In contrast, in HER2/ErbB2-overexpressing cells, PEPD has been reported to exert predominantly inhibitory effects by disrupting ErbB2–Src signaling and promoting receptor internalization and degradation [30,31,32].

These observations suggest that PEPD should not be regarded solely as an enzyme involved in dipeptide degradation but rather as a protein potentially linking proline metabolism with the regulation of key cellular functions, including collagen metabolism, extracellular matrix remodeling, HIF-1α- and TGF-β-dependent signaling, and context-dependent ErbB receptor signaling [29,31,33,34]. However, accumulating evidence suggests that the role of PEPD includes catalytic activity-independent function involving interactions with proteins that control cell survival and death [19,35].

Some studies have demonstrated that PEPD acts as a regulator of p53 transcriptional activity. PEPD directly binds p53, forming a complex present in both the cytoplasm and nucleus, thereby restricting p53 subcellular translocation to mitochondria and inhibiting its phosphorylation. Consequently, PEPD regulates both transcription-dependent and transcription-independent functions of p53 [19,35].

Importantly, PEPD enzymatic activity is not required either for p53 binding or for the regulation of p53 functions. It has been reported that only approximately 6% of the total cellular PEPD pool is involved in p53 binding, whereas more than half of the cellular p53 pool remains associated with PEPD [35]. These observations indicate that PEPD may function as an important regulator of p53 availability, preventing the uncontrolled activation of p53 in unstressed cells. Sequestration of p53 occurs through direct protein–protein interaction, allowing PEPD to compete with mouse double minute 2 homolog (MDM2) for p53 binding. As a result, PEPD prevents MDM2-mediated ubiquitination of p53. Further studies have demonstrated that dissociation of the PEPD–p53 complex by ROS-induced stress activates both transcription-dependent and transcription-independent functions of p53, ultimately leading to growth arrest and apoptotic cell death [35].

From the perspective of the present study, it is particularly important that cell death following PEPD loss does not appear to result from a deficiency of proline or other products generated by PEPD-catalyzed reactions. Yang et al. demonstrated that neither glycyl-proline nor free proline prevented cell death induced by PEPD silencing, supporting the hypothesis that the pro-survival function of PEPD in this experimental context may be independent of its enzymatic activity. The authors also observed the activation of apoptotic pathways, including an increased expression of pro-apoptotic proteins, cytochrome c release, and the activation of caspases [35].

Yang et al. established that intracellular PEPD directly interacts with p53 and limits both its transcription-dependent and transcription-independent functions independently of PEPD catalytic activity [35]. Importantly, their study also demonstrated cell loss following CRISPR/Cas9-mediated PEPD targeting and provided extensive mechanistic validation using siRNA-mediated PEPD depletion, p53-deficient cellular models, pharmacological inhibition of p53, and restoration of intracellular PEPD. Thus, the PEPD–p53 regulatory relationship itself is already well-established [35,36,37].

This framework was subsequently extended by Yang et al. to multiple breast cancer models, including p53-proficient MCF7 cells [36]. Using an orthogonal siRNA-based approach, those authors showed that PEPD depletion markedly reduced MCF7 cell survival and activated p53-dependent downstream responses without increasing the total p53 abundance. They further documented mitochondrial dysfunction and apoptosis-related responses following PEPD depletion [36]. Thus, PEPD-dependent activation of wild-type p53 in MCF7 cells is not itself a novel observation of the present study. Rather, the present work addresses a different experimental context: the early population-level phenotype associated with nuclease-based PEPD targeting in heterogeneous MCF7 cultures before a genetically defined edited population can be established.

Evidence obtained in MCF7 breast cancer cells further suggests that this regulatory relationship may operate in a cell-context-dependent manner. Previous studies in this model have associated increased PEPD abundance and enhanced PEPD–p53 complex formation with the attenuation of p53-dependent signaling [35,37]. However, the early cellular response associated with direct targeting and reduction in endogenous PEPD has not been characterized in detail in MCF7 cells. This question is particularly relevant because MCF7 cells lack functional caspase-3, placing greater importance on alternative effector mechanisms, including caspase-7-mediated proteolysis [38,39]. An important methodological consideration in this context is that nuclease-based CRISPR/Cas9 editing is itself capable of eliciting a DNA-damage response. Cas9-induced genomic double-strand breaks can activate p53-dependent signaling and cell-cycle arrest, making this issue particularly relevant in p53-proficient MCF7 cells. Consequently, changes in p53-, MDM2-, and apoptosis-related proteins following PEPD-directed Cas9 cleavage cannot automatically be attributed exclusively to reduced PEPD abundance unless a matched genomic-cutting control is included. This potential confounding effect was therefore considered when interpreting the present population-level observations [40,41]. Therefore, the aim of the present study was not to re-establish the PEPD–p53 mechanism itself, but to characterize the early population-level response associated with transient CRISPR/Cas9-mediated targeting of PEPD in heterogeneous MCF7 cultures. Three independent sgRNAs targeting distinct regions of the PEPD gene were used to determine whether reduced population-average PEPD abundance and enzymatic activity are accompanied by a concordant pattern involving p53/MDM2-related signaling and apoptosis-related proteins.

## 2. Results

### 2.1. Detection of GFP Expression Following CRISPR/Cas9 Plasmid Transfection in MCF7 Cells

To document detectable expression of the CRISPR/Cas9 plasmids, MCF7 cells were transfected with three independent all-in-one constructs targeting the human PEPD gene. Two versions of each construct were used: one carrying the puromycin (Puro) resistance gene and the other expressing green fluorescent protein (GFP).

Representative brightfield images obtained 72 h after transfection with the CRISPR/Cas9-Puro constructs demonstrated comparable cell morphology in all three transfected populations. In parallel, transfection with the corresponding GFP-expressing constructs resulted in detectable GFP-positive cells for each of the three sgRNA sequences. GFP-positive cells were detected following transfection with each of the three PEPD-targeting constructs. These representative images document successful plasmid expression but were not used to determine transfection or genome-editing efficiency (Supplementary Figure S1).

These observations documented detectable plasmid expression for all three constructs. Because the images were representative and were not acquired using a validated quantitative imaging protocol, they do not provide an estimate of transfection or genome-editing efficiency.

### 2.2. CRISPR/Cas9-Mediated PEPD Targeting Is Associated with Reduced PEPD Activity and a Lower Representative PEPD Protein Signal

MCF7 cells were transfected with three independent CRISPR/Cas9 plasmids targeting the human *PEPD* gene, as described in Section 4.4. Because puromycin selection did not result in a stable, genetically defined population with confirmed PEPD disruption, subsequent biochemical analyses were performed using heterogeneous cultures collected at the indicated time points. These cultures contained transfected, potentially edited, and untransfected cells. In addition, biochemical samples included both adherent cells and cells recovered from the culture medium after detachment during incubation. Representative bulk Western blots showed lower PEPD protein signal in populations transfected with the PEPD-targeting constructs, with the apparent magnitude varying among sgRNAs and time points. Because the samples consisted of heterogeneous cell populations, the observed reduction does not indicate complete PEPD depletion in all cells and cannot be used to infer the proportion of successfully edited cells. Accordingly, the present study provides phenotypic evidence of PEPD-directed perturbation at the protein and enzymatic-activity levels.

The apparent discrepancy between the proportion of visibly GFP-positive cells in representative fluorescence images and the magnitude of PEPD reduction observed in bulk biochemical analyses cannot be resolved by the present experiments. The microscopy and biochemical analyses were performed using different vector variants, experimental formats, and sampling strategies, precluding direct quantitative comparison between these datasets. Moreover, Western blot analysis was performed on the total recovered cell populations, including both adherent and detached cells, whereas fluorescence microscopy represents only adherent cells within selected microscopic fields.

To assess short-term effects associated with plasmid delivery and expression of the corresponding empty vector backbones, MCF7 cells transfected with the empty-vector variants were analyzed using the MTT assay described in Section 4.6. No statistically significant change in MTT reduction was detected relative to the untreated reference cells (Supplementary Figure S2), indicating that empty-vector exposure did not measurably alter metabolic activity under the conditions tested. These controls, however, do not reproduce sgRNA-directed Cas9-mediated genomic cleavage and therefore do not constitute matched genomic-cutting controls for the PEPD-targeting constructs.

PEPD activity was assessed 24 h and 72 h after transfection. Since PEPD enzymatic activity was not measured in the empty-vector cultures, therefore, the activity comparisons shown in Figure 1 use untreated MCF7 cultures as the reference population. In all three groups treated with CRISPR/Cas9, a reduction in PEPD activity was observed compared to the control MCF7^WT^ cells. The most pronounced effect was observed in cells transfected with the sgPEPD-2 plasmid, where activity reached approximately 50% of the value of the control cells (Figure 1A).

Representative Western blot analysis showed lower average PEPD abundance in the heterogeneous populations transfected with each of the three PEPD-targeting constructs (Figure 1B). Because the observations were derived from representative blots without independent biological replication suitable for statistical inference, numerical effect sizes and claims of statistical significance are not reported. All three sgRNAs showed a directionally concordant reduction in the PEPD signal, consistent with the independently replicated decrease in prolidase activity, although the apparent magnitude varied among constructs and time points.

To visualize plasmid-expressing cells and the distribution of PEPD immunofluorescence within the heterogeneous cultures, microscopy was performed using the corresponding GFP-expressing CRISPR/Cas9 constructs. Representative fluorescence images showed heterogeneous PEPD immunoreactivity within the studied cells (Supplementary Figure S3). Some GFP-positive cells displayed relatively weak PEPD staining; however, variability was also present among the GFP-negative cells. Because the images were not acquired for quantitative single-cell analysis, they are presented only as qualitative visualization and are not used to estimate the magnitude or specificity of PEPD reduction.

### 2.3. Exploratory p53- and MDM2-Related Protein Pattern Following PEPD-Directed CRISPR/Cas9 Targeting

To examine p53- and MDM2-related responses, p53 and MDM2 protein signals were assessed by representative Western blotting, with exploratory immunofluorescence used only for qualitative visualization of marker distribution.

Representative Western blot analyses showed higher p53 and MDM2 signals in the PEPD-targeted heterogeneous populations than in the untreated MCF7 reference cultures. The direction of the observed changes was similar across the three sgRNAs, although the apparent signal intensity varied among constructs and time points (Figure 2). Because these observations were not supported by independent biological replicates suitable for inferential analysis, they are described qualitatively and do not provide statistically supported effect sizes or temporal trajectories.

Exploratory immunofluorescence microscopy was additionally performed to visualize marker distribution within heterogeneous cultures. Because field selection, acquisition, and single-cell analysis were not standardized for quantitative comparison, these images are presented in the Supplementary Materials solely as qualitative visualization.

Representative fluorescence images showed heterogeneous p53 and MDM2 immunoreactivity and nuclear-associated p53 fluorescence in some cells. Because subcellular fluorescence was not quantified across predefined fields and independent experiments, the images do not establish p53 nuclear translocation or transcriptional activation (Supplementary Figure S4).

Taken together, these observations identify a qualitative p53/MDM2-related protein-expression pattern associated with PEPD-directed CRISPR/Cas9 targeting.

### 2.4. Exploratory Apoptosis-Related Protein Pattern Following PEPD-Directed CRISPR/Cas9 Targeting

To examine whether CRISPR/Cas9-mediated PEPD targeting was associated with changes in apoptosis-related proteins, the expression of apoptosis markers was analyzed using Western blot and fluorescence microscopy.

Representative Western blot analyses showed higher cleaved caspase-9 and cleaved PARP-1 signals in the PEPD-targeted heterogeneous populations than in the untreated MCF7 reference cultures (Figure 3).

The direction of the pattern was similar across the three sgRNAs, but the apparent magnitude varied among constructs and time points. Because the blots were not supported by independent biological replicates suitable for inferential analysis, the observations are presented qualitatively and no numerical effect sizes or claims of statistical significance are made.

Representative fluorescence images illustrated heterogeneous immunoreactivity for cleaved caspase-9, cleaved caspase-7, and cleaved PARP-1 in cultures containing GFP-positive cells. Because the images were not quantitatively analyzed, they are presented solely as exploratory visualization and were not used to determine marker-positive cell frequencies or to quantitatively compare staining intensity between time points. Qualitative inspection suggested stronger immunoreactivity for cleaved caspase-9 and cleaved PARP-1 in the GFP-positive CRISPR/Cas9-transfected cells than in the MCF7^WT^ control cells (Supplementary Figure S5A,C). Similarly, activated caspase-7, the principal effector caspase in MCF7 cells, showed qualitatively stronger staining in transfected cells. Although staining appeared more prominent at 48 and 72 h after transfection (Supplementary Figure S5B), these observations remain descriptive because no quantitative image analysis was performed.

Together, the representative Western blot and microscopy observations formed an internally concordant qualitative apoptosis-related proteolytic pattern following PEPD-directed CRISPR/Cas9 targeting. These data did not establish the initiating mechanism, mitochondrial membrane permeabilization, apoptosome formation, or functional dependence on p53. Accordingly, cleaved caspase-9, cleaved caspase-7, and cleaved PARP-1 are interpreted here as an apoptosis-related proteolytic protein pattern rather than as a quantitative demonstration of apoptotic cell death.

### 2.5. Qualitative Persistence of Adherent PEPD-Targeted MCF7 Cells During Prolonged Puromycin Selection and Supplementation

Representative brightfield images documented the maintenance of adherent MCF7 populations during prolonged puromycin selection in the absence or presence of L-proline. Untreated MCF7 reference cultures were maintained in DMEM with or without 30 μM L-proline. Cultures transfected with PEPD-targeting CRISPR/Cas9-Puro constructs were subjected to 1.5 μg/mL puromycin in the absence or presence of 30 μM L-proline. Images were acquired 10 days after transfection, following 7 days of puromycin selection and a 10-day observation period. The images provide qualitative documentation of the persistence of adherent selected populations and were not used as a quantitative measurement of viability, apoptosis, or cell death.

Because PEPD participates in the release and recycling of proline from imidodipeptides, we examined whether supplementation with proline or related metabolites altered the qualitative persistence of adherent PEPD-targeted MCF7 cells during prolonged puromycin selection.

MCF7 cells transfected with PEPD-targeting CRISPR/Cas9-Puro constructs were subjected to puromycin selection in the absence or presence of 30 μM L-proline. Representative brightfield images were acquired up to 10 days after transfection. Under all three PEPD-targeting conditions, the number of adherent cells visible during prolonged selection decreased, and L-proline supplementation did not visibly alter this qualitative outcome (Figure 4).

Similar qualitative observations were obtained following supplementation with glycyl-L-proline, glycine, alanine, hydroxyproline, or recombinant PEPD (Supplementary Figure S6).

## 3. Discussion

The present study indicates that CRISPR/Cas9-mediated targeting of PEPD in heterogeneous MCF7 cultures was associated with a partial population-level reduction in PEPD abundance and prolidase activity, together with a qualitative protein-expression pattern involving p53, MDM2, cleaved caspase-9, cleaved PARP-1, and cleaved caspase-7 immunoreactivity. Three sgRNAs targeting distinct regions of the PEPD gene produced directionally concordant responses, despite differences in the apparent magnitude of PEPD reduction. This concordance makes a guide-specific off-target effect less likely, but it does not establish that reduced PEPD abundance was the sole cause of the observed molecular changes. Because transfection and editing efficiency, target-locus genotype, and PEPD abundance were not determined at the single-cell level, the present data do not define a critical threshold of PEPD reduction or the response of individual edited cells.

The potential sensitivity of MCF7 cells to a further decrease in PEPD is of particular interest in light of the observations of Palka and Phang, who reported that basal prolidase activity in MCF7 cells was approximately one-third of that measured in normal skin fibroblasts [42]. A relatively limited basal PEPD pool may reduce the capacity of these cells to tolerate an additional perturbation of PEPD-dependent functions. This interpretation remains hypothetical, because neither the intracellular distribution of PEPD nor the relationship between PEPD abundance and cell fate was assessed in individual cells. Nevertheless, the findings are consistent with the possibility that the biological significance of PEPD in MCF7 cells extends beyond its canonical enzymatic role in the hydrolysis of imidodipeptides containing C-terminal proline or hydroxyproline.

These observations should be considered in the context of the study by Yang et al. [35]. Those authors reported rapid loss of UM-UC-3 cells following CRISPR/Cas9-mediated targeting of PEPD, and in a separate siRNA model, progressive loss of cell survival accompanied by the activation of apoptosis-related pathways. Cell loss after PEPD siRNA treatment was partially attenuated by recombinant wild-type PEPD and by the enzymatically inactive PEPD^^G278D^ mutant; importantly, both proteins entered the cells and partially preserved the intracellular PEPD pool. In contrast, neither Gly-Pro nor proline restored cell survival [35]. These findings support an intracellular, catalysis-independent function of PEPD in that experimental system, but they do not make the models directly equivalent because the present study used a different cell line, a different PEPD-targeting strategy, and a different rescue design.

In the present study, supplementation with proline, Gly-Pro, glycine, alanine, or hydroxyproline did not permit maintenance of the selected population under the conditions tested. This negative result does not establish that the phenotype was independent of PEPD catalytic activity, because supplementation with individual amino acids or Gly-Pro is not equivalent to the restoration of intracellular PEPD catalytic function. Recombinant PEPD likewise did not visibly alter the persistence of adherent cells during prolonged puromycin selection. An adherent selected population could not be maintained during prolonged puromycin exposure under the conditions tested. A definitive rescue experiment would require the re-expression of an sgRNA-resistant PEPD construct or another approach that demonstrably restores the intracellular PEPD pool.

Representative Western blots showed higher p53 signal in PEPD-targeted populations at both analyzed time points. Because the blots were not independently replicated, no quantitative temporal trajectory can be inferred from these observations. This pattern is compatible with an early cellular stress response, but the present experiments do not establish that reduced PEPD was its sole initiating signal or that the subsequent apoptosis-related changes were functionally dependent on p53. Yang et al. demonstrated that PEPD binds directly to the proline-rich domain of p53 and sequesters a substantial fraction of the cellular p53 pool, thereby limiting transcription-dependent and transcription-independent p53 functions [35]. On this basis, partial reduction in intracellular PEPD could decrease the capacity to sequester p53 and increase the fraction available for stress-response signaling. However, the PEPD–p53 interaction, p53 transcriptional activity, phosphorylation, and trafficking were not directly examined here. Moreover, a sequestration model does not by itself explain an increase in total p53 abundance, which may also reflect a DNA-damage response associated with CRISPR/Cas9-mediated cleavage [40,41].

Functional dependence on p53 was not directly tested in the present MCF7 model. Therefore, the increase in p53-related signals and the appearance of apoptosis-related proteins cannot by themselves establish a PEPD→p53→apoptosis causal sequence. Nevertheless, this interpretation is supported by extensive independent mechanistic evidence. Yang et al. demonstrated that siRNA-mediated PEPD depletion reduced survival in p53-proficient cells but not in corresponding p53-deficient HCT116 cells, and that pharmacological inhibition of p53 substantially protected cells from PEPD-depletion-associated loss of survival. Pan-caspase inhibition likewise produced marked protection [35]. Thus, p53 and caspase dependence of the PEPD-depletion phenotype has been demonstrated independently, although confirmation of the same dependency specifically in PEPD-targeted MCF7 cells will require an orthogonal depletion strategy and direct p53 perturbation.

The findings of Yang et al. are particularly informative for interpretation of the present MCF7 observations because their study included MCF7 cells and used siRNA-mediated PEPD depletion, thereby avoiding Cas9-induced genomic double-strand breaks [36]. In that model, PEPD knockdown markedly reduced survival and activated p53-dependent downstream signaling, mitochondrial dysfunction, and apoptosis, but did not increase the total p53 abundance [36]. This distinction is relevant to the present study: the apparent increase in total p53 signal observed in our representative CRISPR/Cas9 experiments may therefore reflect a perturbation-specific component, including a possible Cas9-induced DNA-damage response [40,41], rather than a necessary consequence of PEPD depletion itself. The present data do not allow these contributions to be quantitatively separated.

Representative blots showed an MDM2-related signal pattern compatible with the engagement of p53/MDM2-related signaling. In the established p53–MDM2 feedback system, transcriptionally active p53 can induce MDM2 expression [43]. However, p53 transcriptional activity was not measured, and the available data do not establish temporal dynamics, MDM2-dependent p53 ubiquitination, or functional activation of the p53–MDM2 feedback loop. The observations should therefore be interpreted as a qualitative protein-expression pattern rather than as direct evidence of pathway activation.

Representative Western blot analyses showed cleaved caspase-9 and cleaved PARP-1 signals, whereas exploratory microscopy visualized heterogeneous immunoreactivity for cleaved caspase-9, cleaved caspase-7, and cleaved PARP-1. Considered together, these observations form a qualitative pattern compatible with intrinsic apoptosis-related signaling. Cleaved caspase-9 is consistent with activation of an initiator caspase associated with the mitochondrial pathway [44]; however, mitochondrial outer-membrane permeabilization, cytochrome c release, loss of mitochondrial membrane potential, and apoptosome formation were not measured and cannot be inferred directly. In MCF7 cells, which lack functional caspase-3, caspase-7 can contribute substantially to effector-caspase activity [38,39]. PARP-1 cleavage provides additional evidence of effector-caspase activity [45], but it does not independently establish the initiating mechanism or p53 dependence of the response.

A major limitation is that the Western blot analyses were based on representative blots and were not supported by independent biological replicates suitable for statistical inference. Consequently, the study does not provide statistically supported effect sizes or temporal trajectories for p53, MDM2, cleaved caspase-9, or cleaved PARP-1, and the microscopy does not provide quantitative estimates for cleaved caspase-7. The similar direction of the observations across three PEPD-targeting sgRNAs strengthens the internal consistency of the qualitative pattern, but it should not be described as biological reproducibility. The protein data therefore identify an exploratory pattern associated with CRISPR/Cas9-mediated PEPD targeting rather than quantitatively established changes in abundance.

The microscopy data require similarly cautious interpretation. Representative images showed heterogeneous marker distribution within cultures containing GFP-positive cells. Nuclear-associated p53 fluorescence was visible in some cells, but the experiments were not designed for quantitative single-cell analysis and cannot establish p53 nuclear translocation, marker-positive cell frequency, effect size, or statistical significance. Interpretation is further limited by survivorship bias because detached or severely damaged cells were not retained during fixation and washing, and by apoptosis-associated changes in morphology, membrane permeability, autofluorescence, and nonspecific antibody retention. In addition, microscopy and biochemical experiments used different vector variants, culture formats, and sampling strategies. Western blotting included both adherent and detached cells recovered from the culture, whereas microscopy represented only adherent cells within selected fields. Direct numerical comparison between visibly GFP-positive cells and changes in bulk lysates is therefore not justified.

A critical limitation of the present study is the absence of a matched CRISPR genomic-cutting control. Empty-vector experiments indicate that plasmid delivery and vector expression alone did not measurably reduce MCF7 metabolic activity under the conditions tested, but these controls do not reproduce sgRNA-directed Cas9 cleavage. Although a non-targeting sgRNA would provide an additional control for sgRNA and Cas9 exposure, it would not reproduce a programmed genomic double-strand break. A guide directed against a phenotypically neutral or safe-harbor locus would therefore provide a more stringent cutting control for distinguishing PEPD-dependent signaling from a generic response to Cas9-mediated DNA cleavage. This issue is relevant because CRISPR/Cas9-induced double-strand breaks can activate p53-dependent DNA-damage signaling [40,41]. The use of three independent PEPD-targeting sgRNAs and the directionally concordant reduction in PEPD abundance and enzymatic activity make a guide-specific off-target explanation less likely but do not exclude a common DNA-damage component. Accordingly, the present data support an association between PEPD-directed targeting and the observed molecular phenotype, but cannot attribute that phenotype exclusively to reduced PEPD abundance.

All biochemical analyses were performed in heterogeneous populations containing transfected, potentially edited, and untransfected cells. Western blot and prolidase activity assays therefore reflect the average response of the recovered culture and do not establish complete or biallelic PEPD loss in individual cells. An adherent selected population could not be maintained during prolonged puromycin exposure under the conditions tested. Empty-vector controls address short-term effects of plasmid delivery and vector expression but neither model sgRNA-directed DNA cleavage nor exclude effects of prolonged antibiotic selection. Failure to recover a stable population therefore does not demonstrate that complete PEPD loss is lethal or that PEPD is essential for MCF7 cell survival. Incomplete editing, failure to generate biallelic loss-of-function alleles, selective loss of highly transfected cells, Cas9-induced DNA damage, and reduced fitness associated with PEPD targeting remain plausible, non-mutually exclusive explanations. Validation using an orthogonal PEPD-depletion strategy, together with an appropriate intracellular rescue experiment, will be required to determine whether the observed molecular changes are attributable to reduced PEPD abundance independently of Cas9-mediated DNA cleavage.

In the broader context of cancer biology, PEPD may exert context-dependent functions. Increased prolidase activity has been reported in tumor tissues and in the serum of patients with several malignancies and has been linked to collagen turnover, extracellular-matrix remodeling, invasion, and metastasis [1,2,42,46,47,48]. Separately, the intracellular interaction between PEPD and p53 described by Yang et al. suggests a potential role in regulating cellular stress responses and survival [35]. These functions are not necessarily mutually exclusive. The present observations are consistent with the possibility that intracellular PEPD contributes to p53-related regulation in MCF7 cells, but they do not establish a general dual role of PEPD across cancer types or demonstrate that the same mechanism operates in other cellular contexts.

The possibility that the modulation of intracellular PEPD could influence endogenous p53 activity in tumors retaining functional p53 warrants further investigation. Therapeutic implications are nevertheless premature without confirmation of PEPD-specific causality, definition of p53 dependence, and assessment in genetically defined models. Such studies should incorporate matched CRISPR controls, quantitative assessment of editing efficiency, independent biological replication, single-cell analysis, orthogonal depletion approaches, and validated intracellular rescue.

In conclusion, PEPD-directed CRISPR/Cas9 perturbation using three independent sgRNAs reproducibly reduced population-average PEPD enzymatic activity in heterogeneous MCF7 cultures. Representative immunoblot analyses additionally showed directionally concordant reductions in PEPD protein signal and an exploratory protein-expression pattern involving p53, MDM2, cleaved caspase-9, and cleaved PARP-1.

Editing of the endogenous PEPD locus was not directly confirmed by genomic analysis, and a matched genomic-cutting control was not included. Consequently, the contribution of reduced PEPD abundance cannot be separated from a potential Cas9-associated DNA-damage response. The present study therefore defines an exploratory molecular phenotype accompanying PEPD-directed CRISPR/Cas9 perturbation in MCF7 cells rather than establishing a PEPD-specific p53-dependent apoptotic mechanism.

## 4. Materials and Methods

### 4.1. Cell Culture

Human breast cancer cells, MCF7 (ATCC^®^ HTB-22™, Manassas, VA, USA) were cultured in high-glucose Dulbecco’s modified Eagle medium (DMEM; Gibco, Thermo Fisher Scientific, USA; 4.5 g/L glucose) supplemented with 10% fetal bovine serum (FBS, Qualified; Gibco, Thermo Fisher Scientific, Grand Island, NY, USA), 100 U/mL penicillin, and 100 μg/mL streptomycin (Penicillin–Streptomycin Solution; Gibco, Thermo Fisher Scientific, USA). Cells were routinely passaged upon reaching approximately 80–90% confluence using 0.25% trypsin-EDTA solution (0.25% Trypsin-EDTA Phenol Red; Gibco, Thermo Fisher Scientific, USA).

Cells were maintained in 75 cm^2^ culture flasks (Nunc™, Thermo Fisher Scientific, USA) containing 15 mL of complete growth medium. The culture medium was replaced every 2–3 days. Cell cultures were maintained at 37 °C in a humidified atmosphere containing 5% CO_2_.

For transfection experiments, MCF7 cells were seeded into multi-well culture plates and cultured overnight to reach approximately 30–40% confluence prior to transfection.

### 4.2. CRISPR/Cas9 Plasmids

All-in-One Non-Viral CRISPR/Cas9 vectors targeting the human PEPD gene were obtained from Applied Biological Materials Inc. (ABM, Richmond, BC, Canada). Three independent single-guide RNA (sgRNA) sequences, each containing a 20-bp target-specific sequence directed against distinct regions of the human PEPD gene, were used throughout the study: Target 1, TGTGCAGGCCGGCTCCATCG; Target 2, ACCGAACGCCCAGTGAAAGA; and Target 3, GTGTCATCGATGTTGACACT. Two variants of each construct were employed. For puromycin selection experiments and biochemical analyses, pNV-sgRNA-Cas9-2A-Puro plasmids were used, whereas fluorescence microscopy studies were performed using the corresponding pNV-sgRNA-Cas9-2A-GFP constructs, which allowed for the identification of cells in which transfection was successful. Both plasmid variants were 9361 bp and 9479 bp in length, respectively, and contained the Cas9 nuclease coding sequence under the control of the EF1α promoter, whereas sgRNA expression was controlled by the human U6 promoter. The bacterial selection marker was ampicillin.

No non-targeting or safe-targeting sgRNA construct was included in the experimental design. Accordingly, the empty-vector controls described in Section 4.6 assess effects associated with plasmid delivery and vector expression but do not reproduce sgRNA-directed Cas9-mediated genomic cleavage. Three independent sgRNAs targeting distinct regions of the PEPD gene were evaluated separately throughout the study to reduce the likelihood that a directionally concordant phenotype reflected a guide-specific off-target event.

### 4.3. Plasmid Amplification, Purification and Quality Assessment

CRISPR/Cas9 plasmids targeting the human PEPD gene were supplied by Applied Biological Materials Inc. (ABM, Richmond, BC, Canada) as transformed Escherichia coli agar stab cultures. Bacteria were propagated in Luria–Bertani (LB) medium supplemented with ampicillin (100 μg/mL). Prior to bacterial inoculation, the LB medium was sterilized by autoclaving and allowed to cool before the addition of ampicillin.

Following overnight bacterial culture, plasmid DNA was isolated using the NucleoBond^®^ Xtra Midi Kit (Macherey-Nagel GmbH & Co. KG, Düren, Germany) according to the manufacturer’s instructions. The concentration and purity of isolated plasmid DNA were determined spectrophotometrically using a SpectroStar Nano microplate reader equipped with an LVis Plate (Software version 6.20; BMG LABTECH, Ortenberg, Germany).

DNA quality was assessed based on absorbance measurements at 260, 280, and 230 nm. The A260/A280 and A260/A230 ratios were used to evaluate protein contamination and the presence of organic contaminants, respectively. Only plasmid preparations exhibiting appropriate purity and concentration were used for subsequent transfection experiments.

### 4.4. Transfection of MCF7 Cells with CRISPR/Cas9 Plasmids

Each sgRNA construct was tested independently in all experiments. MCF7 cells were seeded into Thermo Scientific™ Nunc™ 6-Well Cell Culture-Treated Multidishes (Thermo Fisher Scientific, Waltham, MA, USA) or ibiTreat μ-Plate 96 Well Black plates (ibidi GmbH, Gräfelfing, Germany) and cultured overnight. For experiments involving analyses performed 24, 48, and 72 h after transfection, cells were transfected at approximately 30% confluence to avoid overgrowth and excessive cell density during the experimental period and to ensure comparable culture conditions across all analyzed time points.

Transfection was performed using the Lipofectamine™ 3000 Transfection Kit containing Lipofectamine™ 3000 and P3000™ Reagent (Cat. No. L3000008, Thermo Fisher Scientific, Waltham, MA, USA) according to the manufacturer’s protocol. Three independent CRISPR/Cas9 plasmids targeting different regions of the human PEPD gene were used. For fluorescence microscopy experiments, GFP-expressing CRISPR/Cas9 plasmids were employed, whereas plasmids containing a puromycin resistance gene were used for all other experiments. Because the microscopy and biochemical experiments employed different vector variants, plate formats, and culture-density conditions, the proportion of GFP-positive cells visible in representative microscopy fields was not used to estimate the fraction of cells contributing to the biochemical samples.

For transfection in 6-well plates, 2.5 μg of plasmid DNA and 5 μL of P3000™ Reagent were diluted in Gibco Opti-MEM™ I Reduced Serum Medium (Cat. No. A3635101, Thermo Fisher Scientific, Waltham, MA, USA). In a separate tube, 5 μL of Lipofectamine™ 3000 was diluted in Opti-MEM^®^. For transfection in 96-well plates, 50 ng of plasmid DNA and 0.1 μL of P3000™ Reagent were diluted in Opti-MEM^®^, while 0.2 μL of Lipofectamine™ 3000 was diluted separately in Opti-MEM^®^.

The diluted DNA/P3000™ and Lipofectamine™ 3000 solutions were combined, mixed gently by pipetting, and incubated for 15 min at room temperature to allow the formation of DNA–lipid complexes. Subsequently, 250 μL of the transfection mixture was added to each well of a 6-well plate, whereas 10 μL was added to each well of a 96-well plate.

Following transfection, cells were incubated at 37 °C in a humidified atmosphere containing 5% CO_2_. Twenty-four hours after transfection, the culture medium was replaced with fresh complete DMEM, and cells were further cultured until the indicated experimental time points.

For puromycin selection experiments, cells were transfected at higher confluence (70–80%), in accordance with the manufacturer’s recommendations, to ensure the optimal survival of transfected cells during subsequent antibiotic selection.

### 4.5. Puromycin Selection and Amino Acid Supplementation

To evaluate the consequences of transfection with PEPD-targeting CRISPR/Cas9 constructs, MCF7 cells were transfected with CRISPR/Cas9 plasmids containing a puromycin resistance gene, as described in Section 4.4. Twenty-four hours after transfection, the culture medium was replaced with fresh complete DMEM supplemented with puromycin (1.5 μg/mL). The puromycin concentration was established in preliminary dose–response experiments.

To explore whether extracellular supplementation altered the persistence of adherent PEPD-targeted cells during prolonged puromycin selection, cultures were supplemented with L-proline, glycyl-L-proline (Gly-Pro), glycine, alanine, or hydroxyproline at a final concentration of 30 μM. Recombinant PEPD was added at a final concentration of 100 nM. The concentrations of proline, hydroxyproline, glycine, alanine (30 μM), and recombinant PEPD (100 nM) were selected based on our previous studies investigating the role of prolidase-dependent proline metabolism in breast cancer cells. Within each PEPD-targeted condition, cultures maintained in puromycin-containing medium without additional supplementation served as the supplementation reference.

Puromycin selection was maintained for 7 days, and culture morphology and the persistence of adherent cells were documented by brightfield microscopy for up to 10 days after transfection. Because no quantitative viability or apoptosis assay was performed during this selection experiment, the images were used only to document maintenance of the selected adherent population.

Lower population-average PEPD abundance and enzymatic activity relative to untreated MCF7 reference cultures were used as biochemical evidence that the PEPD-targeting constructs affected the analyzed cultures. These measurements did not establish editing efficiency, target-locus genotype, or biallelic PEPD disruption at the single-cell level.

Supplementation with recombinant PEPD was designed as extracellular protein supplementation and was not considered a validated intracellular rescue experiment.

### 4.6. MTT Assay

To assess whether short-term exposure to the transfection procedure and empty CRISPR/Cas9 vectors affected MCF7 metabolic activity, cells were transfected with the empty vector variants corresponding to those used in the study, as described in Section 4.4. MTT measurements were performed 72 h after transfection. The experiment included (*n* = 3) independent biological experiments, each initiated from an independent cell culture and transfection.

At 72 h after transfection, the culture medium was removed and the MTT assay was performed as described below: 100 μL of PBS and 10 μL of a 12 mM MTT solution (5 mg/mL in PBS) were added to each well, followed by incubation at 37 °C for 30 min. After incubation, the MTT-containing solution was aspirated, and the resulting formazan crystals were dissolved in 100 μL of DMSO. The plates were shaken for 10 min at room temperature, and absorbance was measured at 570 nm using a SPECTROstar^®^ Nano microplate reader (BMG LABTECH, Ortenberg, Germany).

MTT reduction, used as an indicator of cellular metabolic activity, was expressed relative to untreated MCF7WT reference cultures, whose mean absorbance was set at 100%.

### 4.7. Preparation of Cell Homogenates

For biochemical analyses, MCF7 cultures were collected at the indicated time points (24 and 72 h after transfection). At each time point, the culture medium containing detached cells was transferred to 15-mL tubes before collection of the adherent cell fraction. Adherent cells were then washed three times with 1 mL of ice-cold phosphate-buffered saline (PBS), detached using sterile cell scrapers, and pooled with the corresponding medium fraction containing detached cells. The combined cell suspensions were centrifuged at approximately 500× *g* for 10 min at 4 °C. The resulting cell pellets therefore contained both adherent cells and cells that had detached into the culture medium during the incubation period, thereby providing a more representative biochemical sample of the recovered experimental cell population than analysis of the adherent fraction alone.

To remove residual culture medium, the supernatants were discarded, and the cell pellets were resuspended in 3 mL of ice-cold PBS and centrifuged again under the same conditions. After removal of the supernatants, the final cell pellets were resuspended in 50 mM Tris-HCl buffer (pH 7.8).

Cell homogenates were prepared by ultrasonic disruption using a pulse-mode sonicator. Sonication was performed using a Hielscher Ultrasonics GmbH (Teltow, Germany), UP200S ultrasonic processor (200 W, 24 kHz), operated at 60% amplitude, using three 20-s pulses separated by 10-s cooling intervals while the samples were maintained on ice. Samples were maintained in an ice bath throughout the homogenization procedure to prevent overheating and protein degradation.

Homogenates used for PEPD activity measurements were prepared as described above. For Western blot analysis, a protease and phosphatase inhibitor cocktail (Abcam, Cambridge, UK; ab201119) was added to the cell suspension prior to homogenization.

### 4.8. Determination of Total Protein Concentration

Total protein concentration in the cell homogenates was determined according to the method of Lowry et al. [49]. Briefly, 10 μL of cell homogenate was mixed with 140 μL of 50 mM Tris-HCl buffer (pH 7.8) and freshly prepared Lowry reagent consisting of 9.8 mL of 2% sodium carbonate (Na_2_CO_3_), 100 μL of 0.1% copper (II) sulfate (CuSO_4_), and 100 μL of 2% sodium potassium tartrate. The mixtures were vortexed and incubated for 15 min at room temperature.

Subsequently, 100 μL of Folin–Ciocalteu (Sigma-Aldrich, St. Louis, MO, USA) reagent diluted 1:1 with distilled water was added to each sample. The reaction mixtures were incubated for an additional 40 min at room temperature to allow for color development. A blank sample containing 10 μL of distilled water and 140 μL of 50 mM Tris-HCl buffer (pH 7.8) was prepared and analyzed in parallel. Absorbance was measured using a SPECTROstar^®^ Nano spectrophotometer (BMG LABTECH, Ortenberg, Germany) at 750 nm.

Protein concentrations were calculated from a standard curve prepared using bovine serum albumin (BSA; Sigma-Aldrich, St. Louis, MO, USA) standards of known concentrations and expressed as μg/μL. All measurements were performed in triplicate, and the results were expressed as mean values.

### 4.9. PEPD Activity Assay

PEPD activity was determined in cell homogenates according to the method described by Myara et al. [50] based on the colorimetric determination of proline released from the synthetic substrate glycyl-L-proline (Gly-Pro; Sigma-Aldrich, St. Louis, MO, USA). Since PEPD is a manganese-dependent metalloprotease, enzyme activation was achieved by preincubation with manganese ions.

Briefly, 10 μL of cell homogenate was mixed with 40 μL of Tris-HCl buffer (pH 7.8) and 50 μL of 2 mM MnCl_2_. The reaction mixtures were preincubated at 37 °C for 24 h. Subsequently, the enzymatic reaction was initiated by adding 50 μL of Gly-Pro solution (94 mM) and 50 μL of Tris-HCl buffer (pH 7.8). Samples were incubated for 1 h at 37 °C.

The enzymatic reaction was terminated by the addition of 0.45 M trichloroacetic acid (TCA). Blank samples were prepared by adding TCA immediately after the addition of Gly-Pro substrate. The samples were centrifuged at approximately 3000× *g* for 10 min, and 1 mL of freshly prepared Chinard reagent, consisting of 25 g ninhydrin dissolved in 600 mL glacial acetic acid and 400 mL of 6 M orthophosphoric acid [51], was added to the collected supernatants. The mixtures were heated at 90 °C for 10 min to allow for color development resulting from the reaction of Chinard reagent with free proline. Absorbance was measured at 515 nm using a SPECTROstar^®^ Nano spectrophotometer (BMG LABTECH, Ortenberg, Germany).

Proline concentrations were calculated from a standard curve prepared using known concentrations of L-proline. PEPD activity was normalized to total protein content determined by the Lowry method and expressed as nmol of proline released per minute per mg of protein.

### 4.10. Western Blot Analysis

Equal amounts of protein were loaded onto each gel. For PEPD, p53, and MDM2 analyses, exactly 20 μg of total protein was loaded per lane; for cleaved caspase-9 and cleaved PARP-1 analyses, 30 μg was loaded per lane. Proteins were separated by SDS-PAGE using 10% polyacrylamide gels at a constant voltage of 120 V. A molecular weight marker (Perfect Tricolor Protein Ladder; EURx, Gdańsk, Poland) was included in each gel to estimate the molecular weights of the separated proteins. The approximate molecular masses used for band identification were 56 kDa for PEPD, 53 kDa for p53, approximately 89–90 kDa for MDM2, approximately 35 kDa for cleaved caspase-9, approximately 116 kDa for full-length PARP-1, approximately 89 kDa for cleaved PARP-1, and 42 kDa for β-actin. Uncropped source images containing the molecular-weight marker lanes and the corresponding loading-control blots are provided in the supplementary source data.

For caspase-9, the antibody used in the present study was directed against the cleavage-associated form, and full-length caspase-9 was not assessed. In contrast, the PARP-1 antibody used for Western blotting detected both full-length PARP-1 (~116 kDa) and the cleaved PARP-1 fragment (~89 kDa), allowing both molecular species to be visualized on the same blot. Because the Western blot analyses were representative and were not supported by independent biological replicates suitable for inferential analysis, the relative intensities of the full-length and cleaved PARP-1 bands were interpreted descriptively and were not used to calculate a statistically supported cleavage ratio.

Following transfer, membranes were blocked with 5% non-fat dry milk (Santa Cruz Biotechnology, Dallas, TX, USA) prepared in TBST for 1 h at room temperature. Membranes were then washed four times for 15 min each with TBST and incubated overnight at 4 °C with primary antibodies diluted in 5% bovine serum albumin (BSA; Sigma-Aldrich, St. Louis, MO, USA) prepared in TBST. The primary antibodies used in this study are summarized in Table 1.

Following incubation with the primary antibodies, membranes were washed four times for 15 min each with TBST and subsequently incubated for 1 h at room temperature with appropriate horseradish peroxidase (HRP)-conjugated secondary antibodies diluted 1:1000 in 5% BSA in TBST. The secondary antibodies used in this study are summarized in Table 2.

After incubation, membranes were washed four times for 15 min each with TBST.

Protein bands were visualized using Amersham™ ECL Detection Reagents (Cytiva, Marlborough, MA, USA) and detected using the Alliance Imaging System (version 15.08d; Uvitec Ltd., Cambridge, UK). Band intensities were also quantified using the Alliance Imaging System software. Signals corresponding to the analyzed proteins were normalized to the respective β-actin signal shown in Figure 1. The normalized values were used exclusively for descriptive evaluation of the representative Western blots and were not subjected to statistical analysis.

### 4.11. Immunofluorescence Staining and Microscopy

MCF7 cells were seeded into black ibiTreat μ-Plate 96-well plates (ibidi GmbH, Gräfelfing, Germany) and transfected with GFP-expressing CRISPR/Cas9 plasmids targeting the PEPD as described in Section 4.4. Immunofluorescence analyses were performed 24, 48, and 72 h after transfection.

Cells were fixed with 3.7% formaldehyde for 15 min at room temperature and washed with phosphate-buffered saline (PBS). Subsequently, cells were permeabilized with 0.1% Triton X-100 for 10 min and washed twice with PBS. Non-specific binding sites were blocked with 3% fetal bovine serum (FBS) for 30 min at room temperature.

Following blocking, cells were incubated overnight at 4 °C with primary antibodies against PEPD, p53, MDM2, cleaved caspase-9, cleaved caspase-7, and cleaved PARP-1. The antibodies used for immunofluorescence staining are summarized in Table 3.

After incubation, cells were washed three times with PBS and incubated for 1 h at room temperature in the dark with Alexa Fluor 594-conjugated secondary antibodies. The secondary antibodies used in this study are summarized in Table 4.

Following incubation with secondary antibodies, cells were washed three times with PBS and stained with Hoechst 33342 at a final concentration of 10 μg/mL for nuclear visualization. Fluorescence images were acquired using a Pathway 855 fluorescent microscope (BD Biosciences, Franklin Lakes, NJ, USA).

Green fluorescent protein (GFP) expression was used to identify successfully transfected cells. GFP expression was used only as a qualitative indicator of detectable plasmid expression and was not used to determine transfection efficiency, genome-editing efficiency, or the proportion of cells with PEPD locus disruption. Representative images were used to illustrate GFP expression and the distribution of the analyzed immunofluorescence signals within heterogeneous cultures.

Fluorescence images were acquired using a BD Pathway 855 equipped with an Olimpus 40×, using AttoVision software (version 2.0; BD Biosciences, Franklin Lakes, NJ, USA). Brightness and contrast were adjusted linearly and uniformly across comparable images, without local or selective processing. Enlarged insets were cropped from the indicated regions, and scale bars were generated from the original image calibration.

Representative images were acquired. The microscopy experiments were designed as exploratory visualization rather than quantitative single-cell analyses. No predefined random-field selection, automated cell segmentation, standardized background correction, quantitative comparison of GFP-positive and GFP-negative populations, predefined cell number, or statistical analysis across independent biological replicates was performed. Accordingly, the images were not used to estimate transfection or editing efficiency, marker-positive cell frequency, effect size, or statistical significance.

### 4.12. Statistical Analysis

Statistical analyses were performed using GraphPad Prism 9 (GraphPad Software version 9.0.2, San Diego, CA, USA). PEPD activity data are presented as individual biological values together with the mean ± SD from three independent biological experiments. Two-way ANOVA was performed with treatment group (untreated reference, sgPEPD-1, sgPEPD-2, and sgPEPD-3) and time (24 and 72 h) as fixed factors, including the treatment × time interaction. Dunnett’s multiple-comparisons test was subsequently used for prespecified comparisons of each PEPD-targeting condition with the corresponding untreated reference group. MTT data are presented as mean ± SD from *n* = 3 independent biological experiments and were analyzed using one-way ANOVA followed by Dunnett’s multiple-comparisons test. A value of *p* < 0.05 was considered statistically significant. Western blot and immunofluorescence observations were not supported by independent biological replicates suitable for inferential statistical analysis. Densitometric values from representative Western blots were used only to aid qualitative inspection and were not treated as independent observations, effect-size estimates, or evidence of statistical significance.

## Figures and Tables

**Figure 1:**
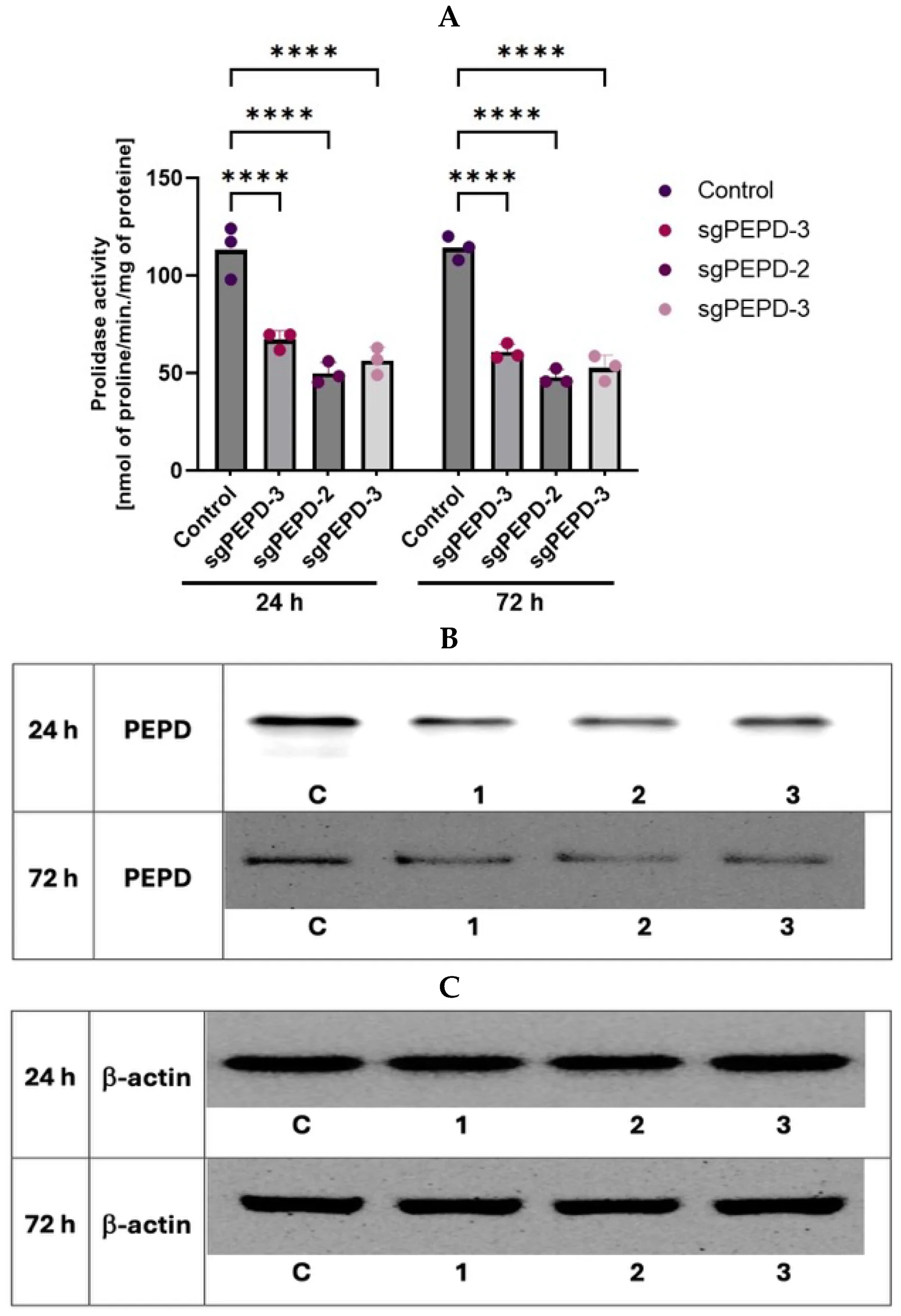
Transfection of MCF7 cells with CRISPR/Cas9 constructs targeting the *PEPD* gene leads to a reduction in PEPD activity and expression. (**A**) PEPD activity in MCF7 cells measured 24 and 72 h after transfection with three independent CRISPR/Cas9 constructs targeting the human *PEPD* gene (sgPEPD-1, sgPEPD-2, and sgPEPD-3). PEPD activity was normalized to the control MCF7^WT^ cells. Data are presented as the mean ± SD from three independent biological experiments. Statistical analysis was performed using two-way ANOVA with treatment and time as fixed factors, including their interaction, followed by Dunnett’s multiple-comparisons test against the corresponding untreated reference group. **** *p* < 0.0001 relative to the MCF7^WT^ control cells. Western blot analysis of PEPD (**B**) and β-actin (**C**) expression in the control MCF7^WT^ cells and cells transfected with CRISPR/Cas9 constructs targeting the *PEPD* gene: sgPEPD-1 (1), sgPEPD-2 (2), and sgPEPD-3 (3). β-Actin was used as a loading control for all Western blot analysis.

**Figure 2:**
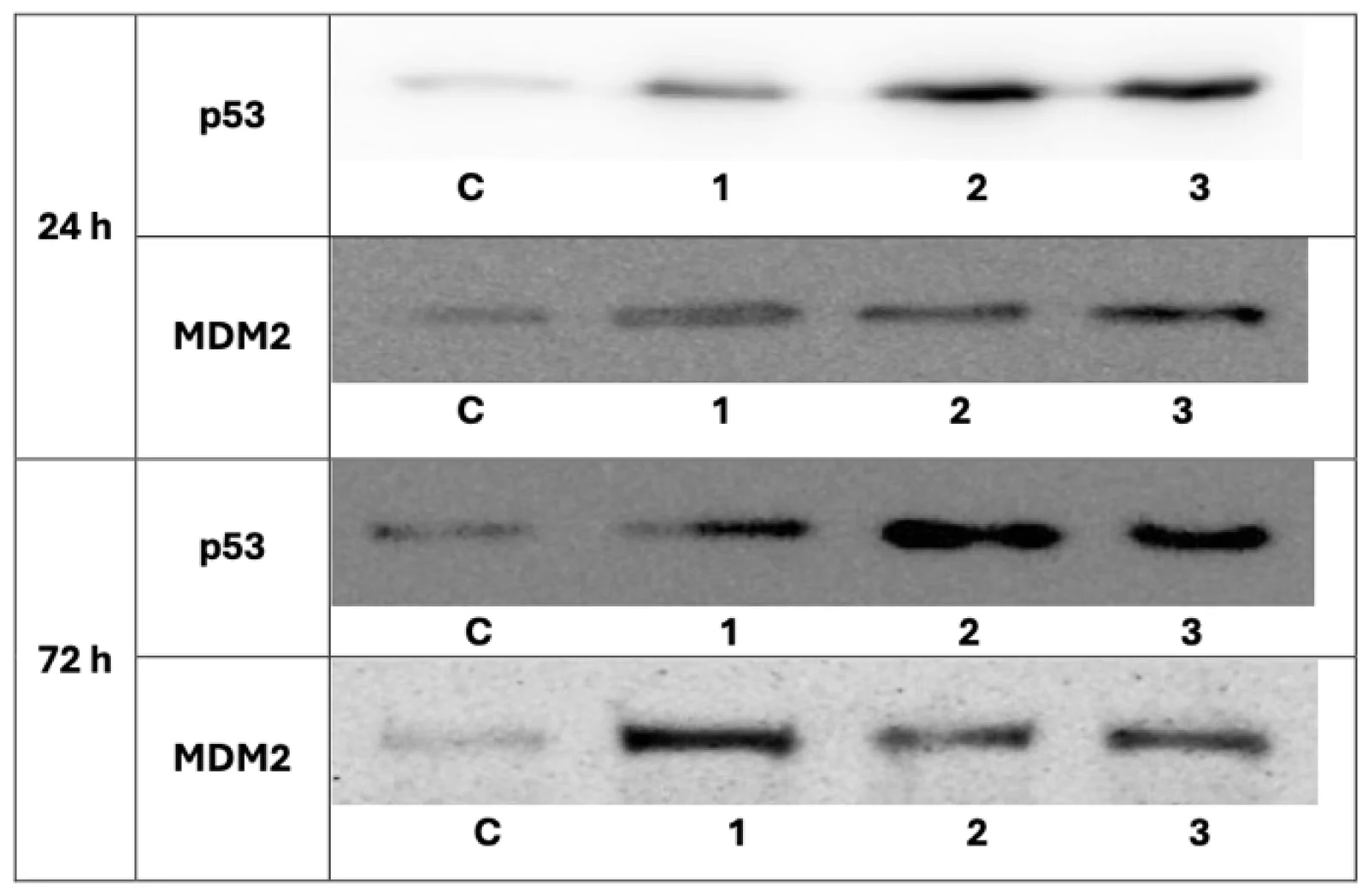
Representative Western blot analysis of p53 and MDM2 protein signals in the untreated MCF7 reference cells (C) and heterogeneous populations transfected with three independent PEPD-targeting CRISPR/Cas9 constructs: sgPEPD-1 (1), sgPEPD-2 (2), and sgPEPD-3 (3), analyzed 24 and 72 h after transfection. Corresponding β-actin loading controls are shown beneath the target-protein blots.

**Figure 3:**
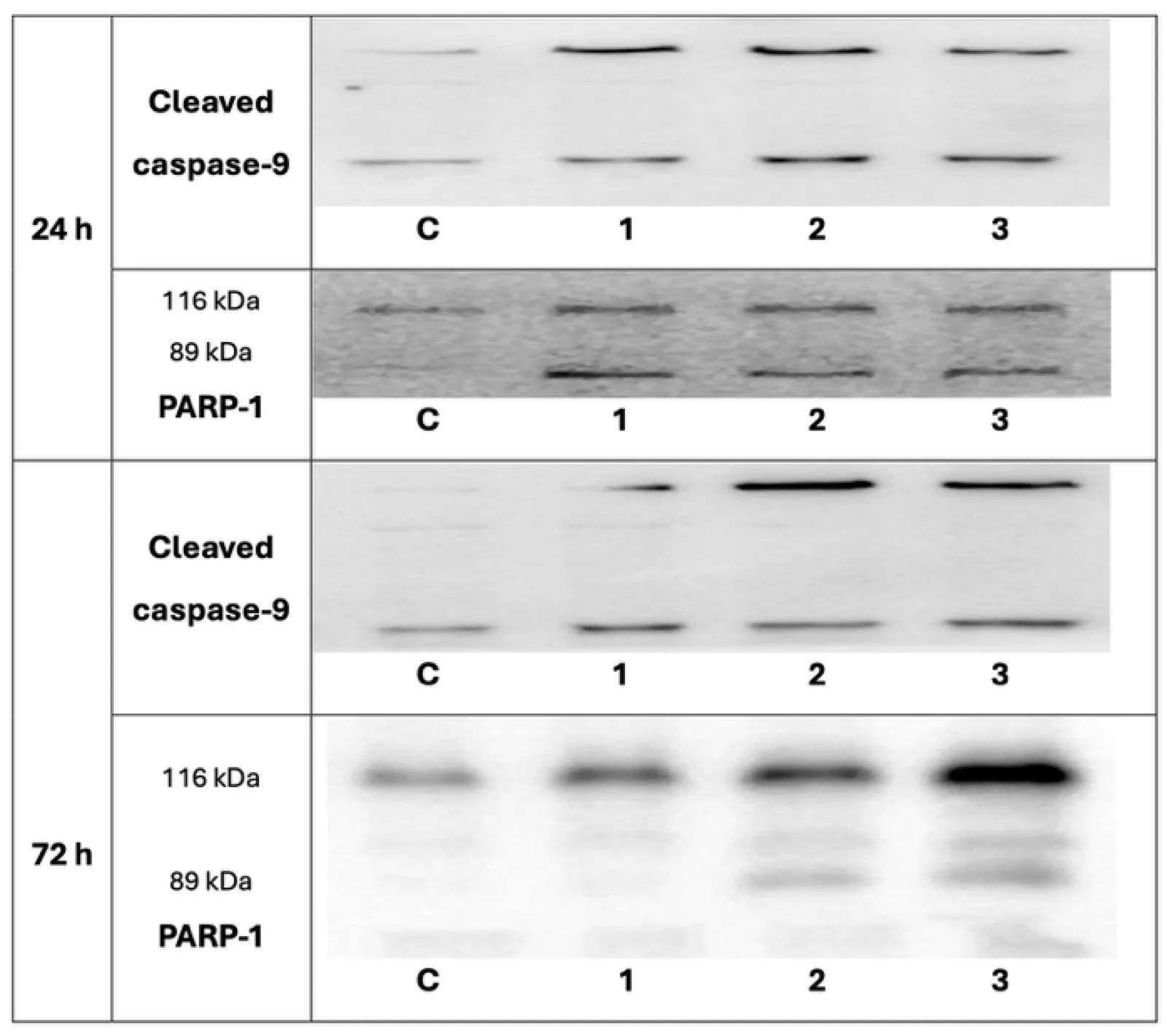
Representative Western blot analysis of cleaved caspase-9 and PARP-1 processing in the untreated MCF7 reference cells (C) and heterogeneous populations transfected with three independent PEPD-targeting CRISPR/Cas9 constructs: sgPEPD-1 (1), sgPEPD-2 (2), and sgPEPD-3 (3), analyzed 24 and 72 h after transfection. For PARP-1, the antibody detected both the full-length protein (~116 kDa) and the cleaved fragment (~89 kDa), both of which are indicated in the blot. Cleaved caspase-9 was identified at approximately 35 kDa; full-length caspase-9 was not assessed. Corresponding β-actin loading controls are shown beneath the target-protein blots.

**Figure 4:**
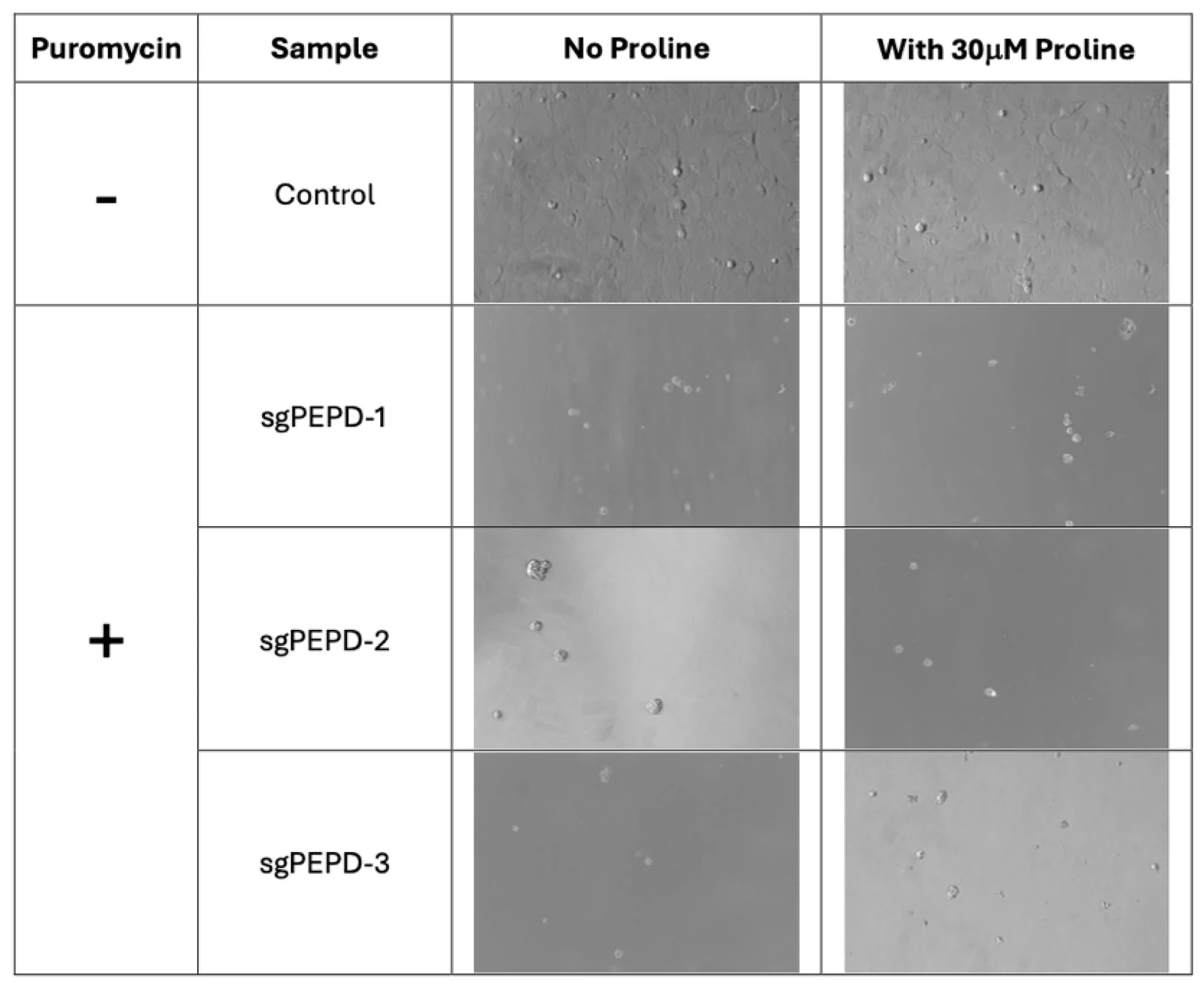
Representative morphology of adherent PEPD-targeted MCF7 cultures during prolonged puromycin selection in the absence or presence of L-proline.

**Table 1:** Primary antibodies used for Western blot analysis.

Primary Antibody	Application	Producer	Catalog No.	Host Species	Dilution
PEPD	WB, IF	Santa Cruz Biotechnology	sc-390042	Mouse	1:200
p53	WB, IF	Santa Cruz Biotechnology	sc-126	Mouse	1:1000
MDM2	WB, IF	Santa Cruz Biotechnology	sc-965	Mouse	1:1000
Cleaved caspase-9	WB, IF	Cell Signaling Technology (Danvers, MA, USA)	20750S	Rabbit	1:1000
PARP-1 (full-length and cleaved)	WB, IF	Cell Signaling Technology	94885S	Rabbit	1:1000
β-actin	WB	Abcam	ab6276	Mouse	1:1000

**Table 2:** Secondary antibodies used for Western blot analysis.

Secondary Antibody	Conjugate	Producer	Catalog No.	Dilution
Goat anti-mouse IgG	HRP	Sigma-Aldrich	A9044	1:1000
Goat anti-rabbit IgG	HRP	Sigma-Aldrich	A9169	1:1000

**Table 3:** Primary antibodies used for immunofluorescence staining.

Primary Antibody	Application	Producer	Catalog No.	Host Species	Dilution
PEPD	WB, IF	Santa Cruz Biotechnology	sc-390042	Mouse	1:100
p53	WB, IF	Santa Cruz Biotechnology	sc-126	Mouse	1:100
MDM2	WB, IF	Santa Cruz Biotechnology	sc-965	Mouse	1:100
Cleaved caspase-9	WB, IF	Cell Signaling Technology	20750S	Rabbit	1:100
Cleaved caspase-7	IF	Cell Signaling Technology	9491S	Rabbit	1:100
Cleaved PARP-1	WB, IF	Cell Signaling Technology	94885S	Rabbit	1:400

**Table 4:** Secondary antibodies used for immunofluorescence staining.

Secondary Antibody	Conjugate	Producer	Catalog No.	Dilution
Goat anti-mouse IgG	Alexa Fluor 594	Thermo Fisher Scientific	A-11005	1:800
Goat anti-rabbit IgG	Alexa Fluor 594	Thermo Fisher Scientific	A-11012	1:800

## Data Availability

The original contributions presented in this study are included in the article and Supplementary Materials. Further inquiries can be directed to the corresponding author.

## Supplementary Materials

The following supporting information can be downloaded at: https://www.mdpi.com/article/10.3390/molecules31193360/s1. Figure S1: Verification of CRISPR/Cas9 transfection efficiency in MCF7 cells. Figure S2: Cytotoxicity of empty CRISPR/Cas9 vectors in MCF7 cells 72 h after transfection. Figure S3: Representative fluorescence microscopy images showing PEPD expression in control MCF7^WT^ cells and cells transfected with three independent CRISPR/Cas9 constructs targeting the *PEPD* gene (sgPEPD-1, sgPEPD-2, sgPEPD-3). Figure S4: Representative fluorescence microscopy images showing the expression and intracellular localization of p53 (A) and MDM-2 (B) in control MCF7^WT^ cells and cells transfected with CRISPR/Cas9 constructs targeting the *PEPD* gene. Figure S5: Representative fluorescence microscopy images showing the expression of cleaved caspase-9 (A), cleaved caspase-7 (B), and cleaved PARP-1 (C) proteins in MCF7^WT^ control cells and cells transfected with CRISPR/Cas9 constructs targeting the *PEPD* gene. Figure S6: Supplementation with proline-related metabolites or recombinant PEPD does not restore the viability of MCF7 cells after PEPD downregulation by CRISPR/Cas9.

## Abbreviations

cleaved PARP-1	Cleaved poly(ADP-ribose) polymerase 1
EGFR	Epidermal growth factor receptor
GFP	Green fluorescent protein
GLUT1	Glucose transporter 1
HER2	Human epidermal growth factor receptor 2
HIF-1α	Hypoxia-inducible factor 1 alpha
MDM2	Mouse double minute 2 homolog
MTT	3-(4,5-Dimethylthiazol-2-yl)-2,5-diphenyltetrazolium bromide
P5C	Δ^1^-Pyrroline-5-carboxylate
PEPD	Prolidase (Peptidase D)
PRODH/POX	Proline dehydrogenase/proline oxidase
Puro	Puromycin
PYCR	Pyrroline-5-carboxylate reductase
ROS	Reactive oxygen species
TGF-β	Transforming growth factor beta
VEGF	Vascular endothelial growth factor
