# Supplementary material for: CRISPR/Cas9-Directed PEPD Targeting Is Associated with Reduced PEPD Activity and an Exploratory p53- and Apoptosis-Related Protein Pattern in MCF7 Breast Cancer Cells
Source: Molecules. 2026 Sep 22;31(19):3360. doi: 10.3390/molecules31193360 (PMC13666405; doi:10.3390/molecules31193360)
Supplement: Supplementary file 1 [file molecules-31-03360-s001.zip › Supplementary Figure S1.pdf]

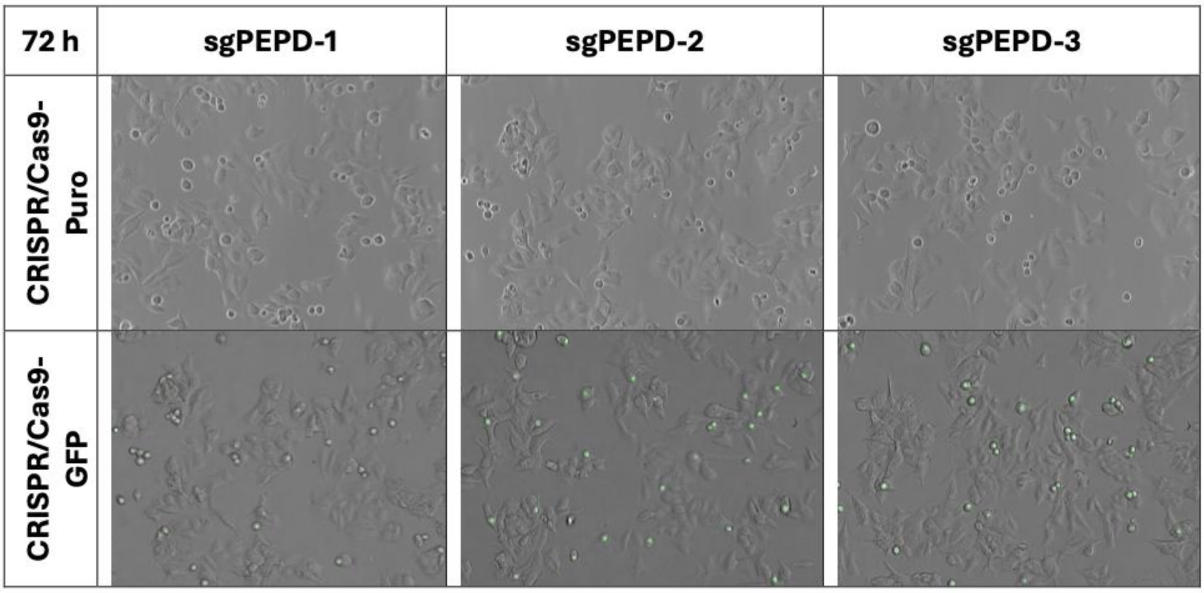

Supplementary Figure S1.

Qualitative detection of GFP reporter expression following CRISPR/Cas9 plasmid transfection in MCF7 cells.

Representative brightfield and fluorescence images of MCF7 cells 72 h after transfection with CRISPR/Cas9 plasmids targeting PEPD. GFP fluorescence confirms detectable expression of the corresponding CRISPR/Cas9-GFP constructs. The images are representative and were not used for quantitative assessment of transfection or genome-editing efficiency.
