# Supplementary material for: CRISPR/Cas9-Directed PEPD Targeting Is Associated with Reduced PEPD Activity and an Exploratory p53- and Apoptosis-Related Protein Pattern in MCF7 Breast Cancer Cells
Source: Molecules. 2026 Sep 22;31(19):3360. doi: 10.3390/molecules31193360 (PMC13666405; doi:10.3390/molecules31193360)
Supplement: Supplementary file 1 [file molecules-31-03360-s001.zip › Supplementary Figure S2.pdf]

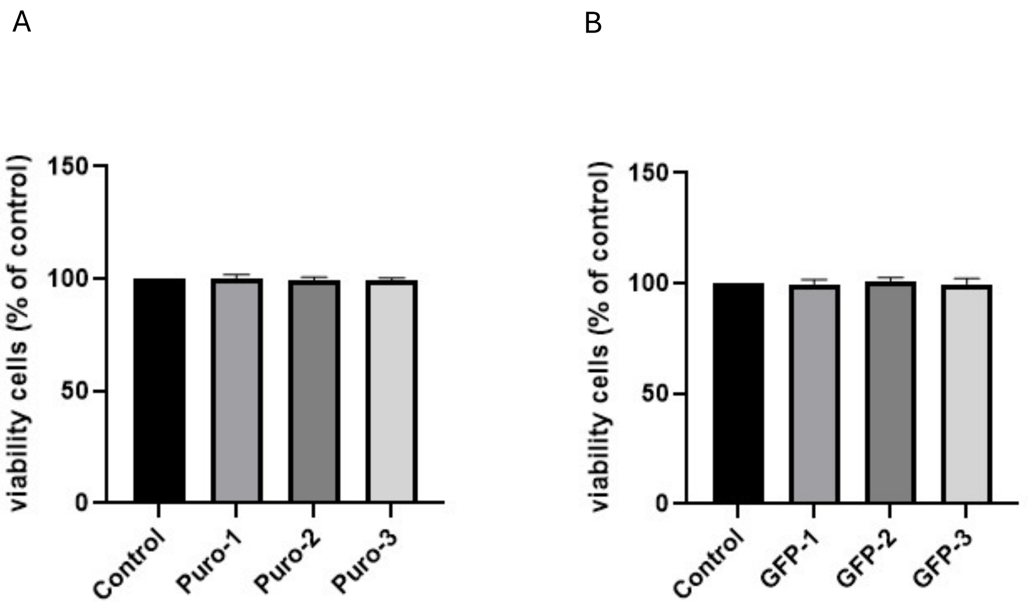

Supplementary Figure S2.

Empty CRISPR/Cas9 vectors do not affect the viability of MCF-7 cells.

(A) Cell viability of MCF7 cells 72 h after transfection with three independent empty CRISPR/Cas9 vectors carrying the puromycin resistance gene (CRISPR/Cas9-Puro). **(B)** Cell viability of MCF7 cells 72 h after transfection with three independent empty CRISPR/Cas9 vectors expressing GFP (CRISPR/Cas9-GFP). Cell viability was determined using the MTT assay and expressed as a percentage of non-transfected control cells (100%). Data are presented as mean ± SD from three independent biological experiments. Statistical analysis was performed using one-way ANOVA followed by Dunnett's multiple comparisons test. No statistically significant differences were observed between control cells and any of the transfected groups.
