# Supplementary material for: CRISPR/Cas9-Directed PEPD Targeting Is Associated with Reduced PEPD Activity and an Exploratory p53- and Apoptosis-Related Protein Pattern in MCF7 Breast Cancer Cells
Source: Molecules. 2026 Sep 22;31(19):3360. doi: 10.3390/molecules31193360 (PMC13666405; doi:10.3390/molecules31193360)
Supplement: Supplementary file 1 [file molecules-31-03360-s001.zip › Supplementary Figure S3.pdf]

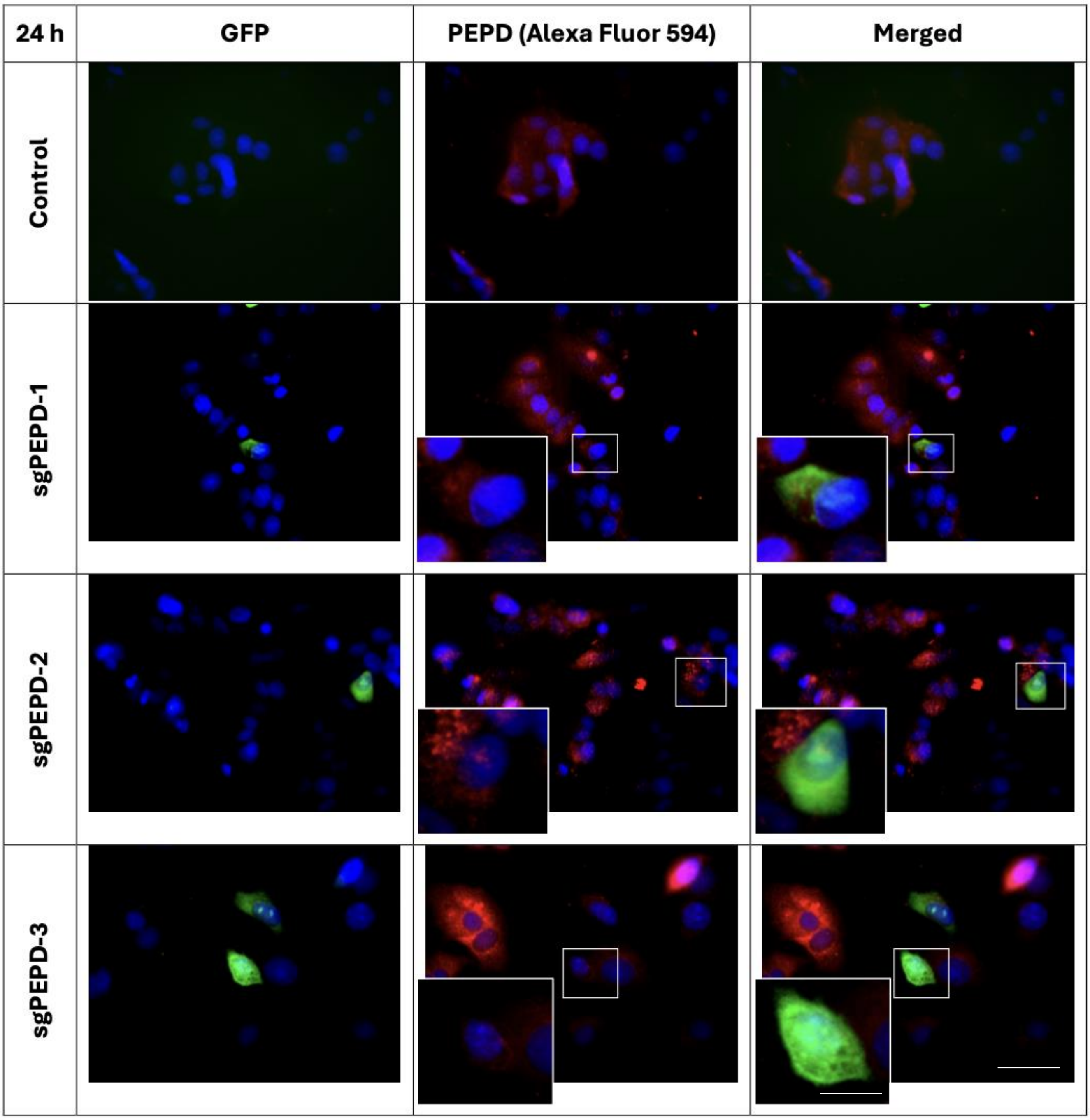
24 h
GFP
PEPD (Alexa Fluor 594)
Merged
Control
sgPEPD-1
sgPEPD-2
sgPEPD-3

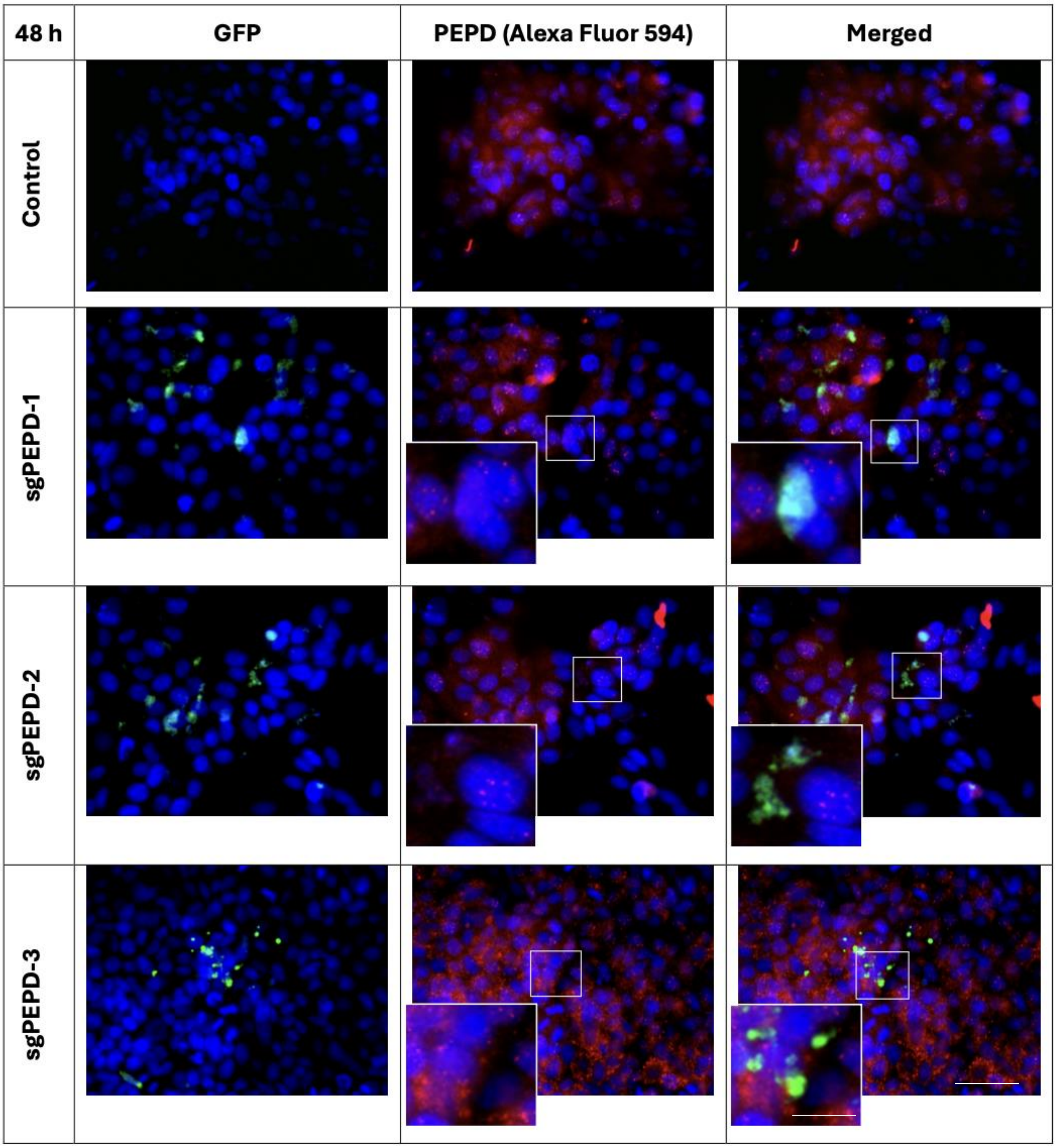
48 h
GFP
PEPD (Alexa Fluor 594)
Merged
Control
sgPEPD-1
sgPEPD-2
sgPEPD-3

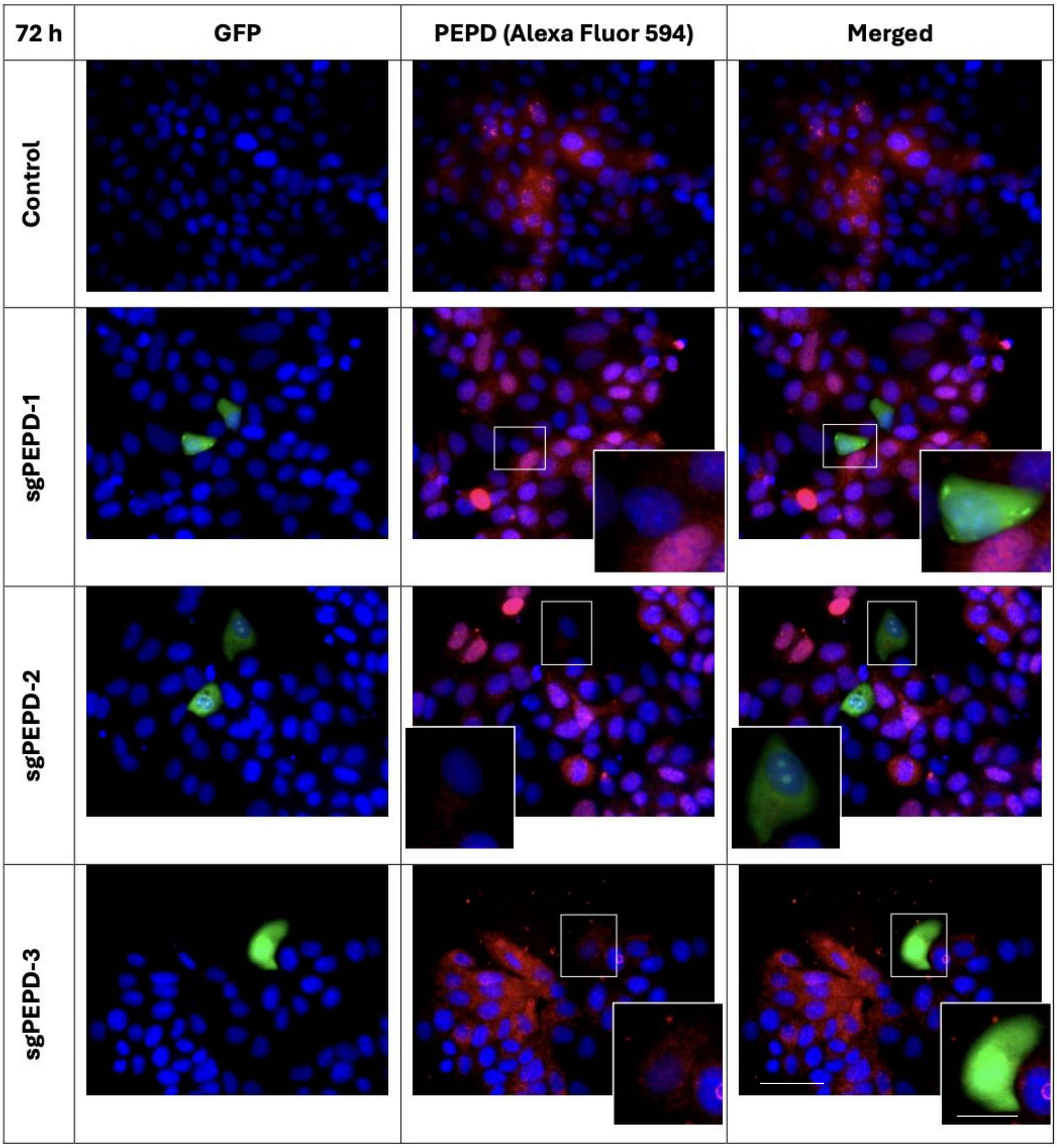

Figure S3. Representative fluorescence microscopy images showing PEPD expression in control MCF7[WT] cells and cells transfected with three independent CRISPR/Cas9 constructs targeting the *PEPD* gene (sgPEPD-1, sgPEPD-2, sgPEPD-3). GFP fluorescence (green) was used to identify successfully transfected cells, while PEPD expression was visualized using secondary antibodies conjugated with Alexa Fluor 594 (red). Cell nuclei were stained with Hoechst 33342 (blue). The merged images illustrate the distribution of GFP expression and PEPD immunofluorescence within the heterogeneous cultures. The images were taken 24, 48, and 72 hours after transfection using an Olympus 40x objective. Representative images illustrate GFP expression and heterogeneous PEPD immunoreactivity after transfection with PEPD-targeting CRISPR/Cas9 constructs. The images were not subjected to

quantitative single-cell fluorescence analysis and should not be interpreted as a measurement of transfection efficiency, genome-editing efficiency, or the magnitude of PEPD depletion. White boxes indicate the regions shown at higher magnification in the corresponding enlarged insets. Insets were generated by cropping the indicated regions from the respective original images and enlarging them for visualization only; no additional image processing was applied specifically to the inset regions. Scale bars: 50 μm in the main images and 10 μm in the enlarged insets.
