# Supplementary material for: CRISPR/Cas9-Directed PEPD Targeting Is Associated with Reduced PEPD Activity and an Exploratory p53- and Apoptosis-Related Protein Pattern in MCF7 Breast Cancer Cells
Source: Molecules. 2026 Sep 22;31(19):3360. doi: 10.3390/molecules31193360 (PMC13666405; doi:10.3390/molecules31193360)
Supplement: Supplementary file 1 [file molecules-31-03360-s001.zip › Supplementary Figure S4.pdf]

A

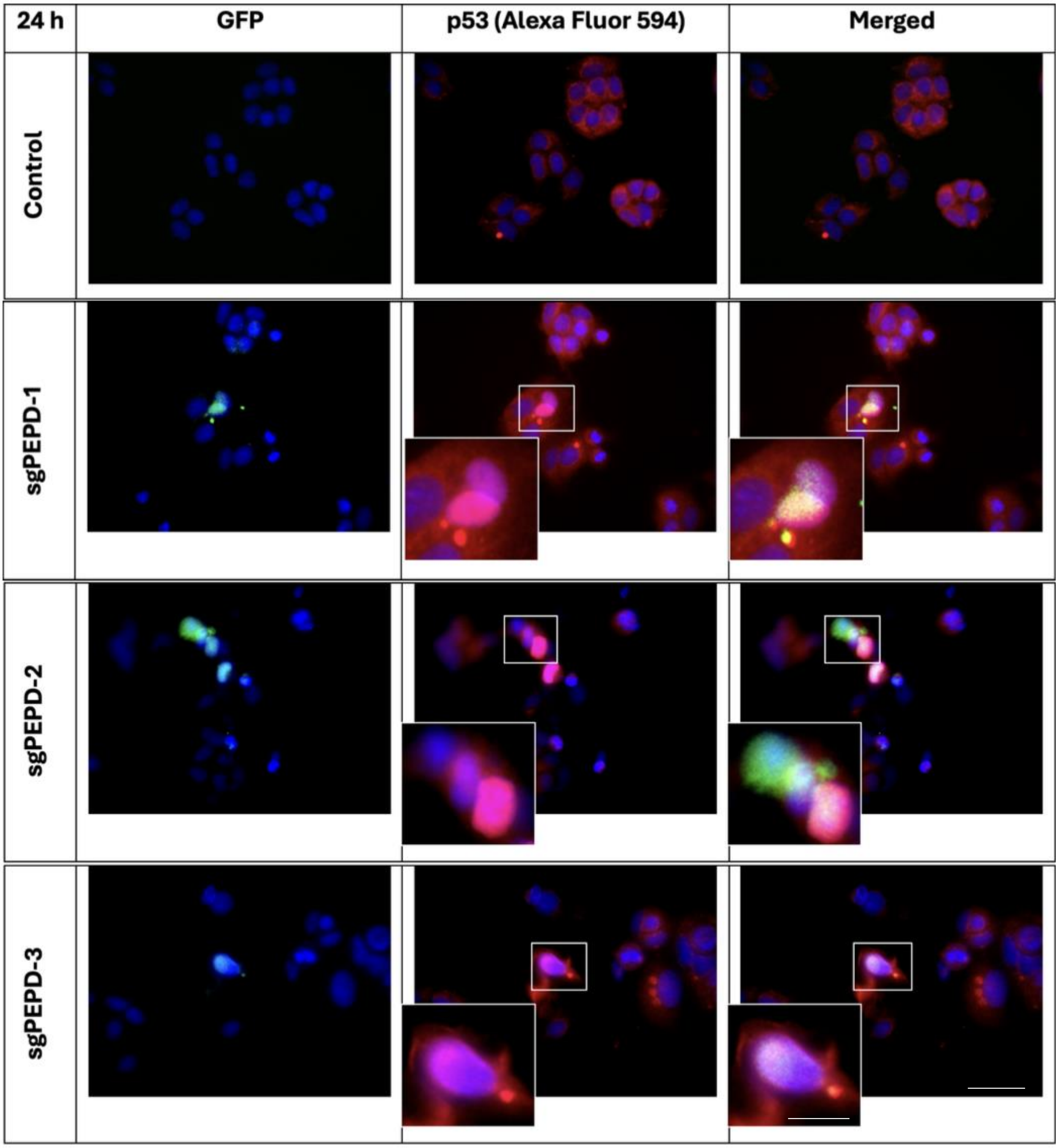
24 h
GFP
p53 (Alexa Fluor 594)
Merged
Control
sgPEPD-1
sgPEPD-2
sgPEPD-3

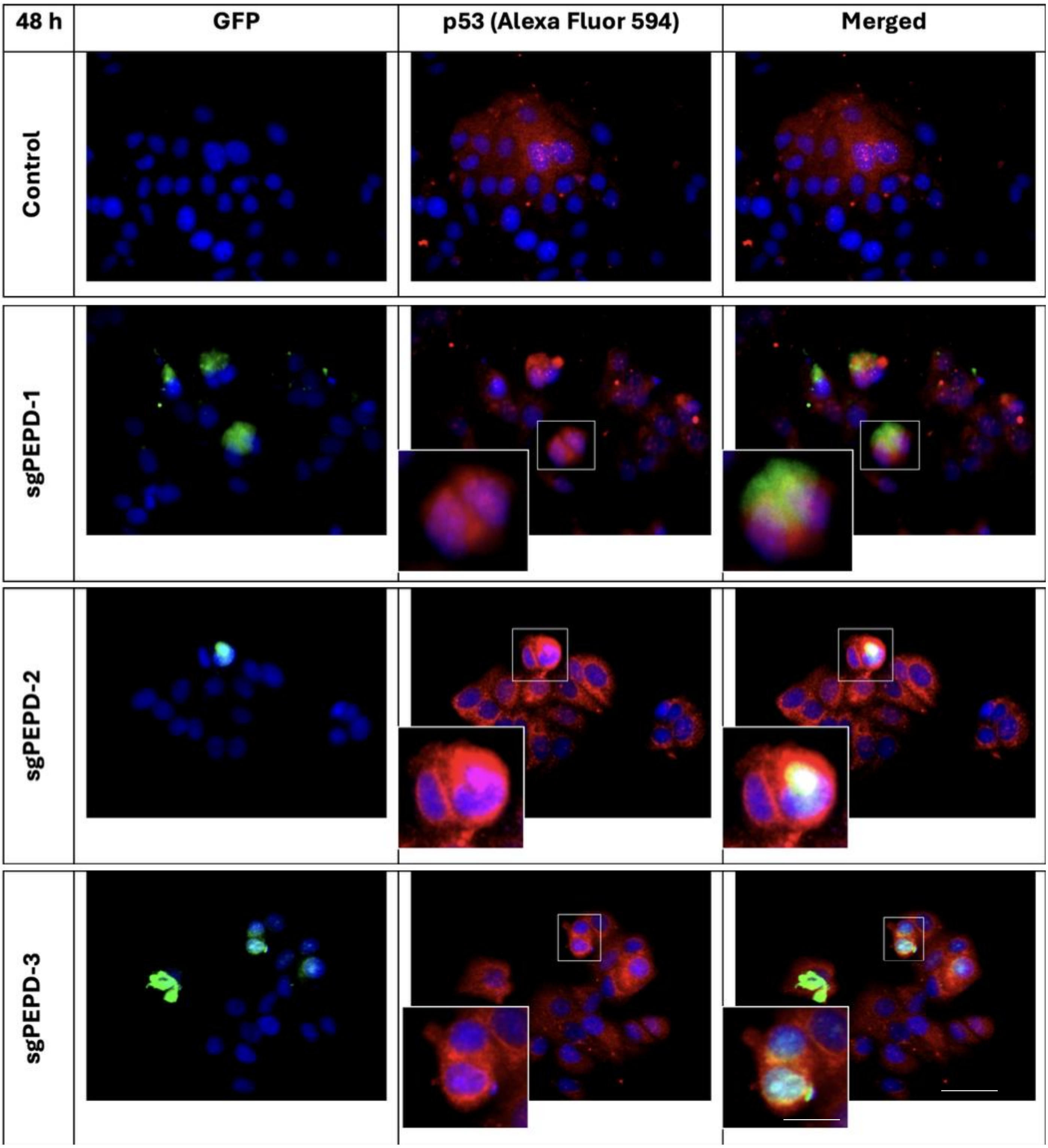
48 h
GFP
p53 (Alexa Fluor 594)
Merged
Control
sgPEPD-1
sgPEPD-2
sgPEPD-3

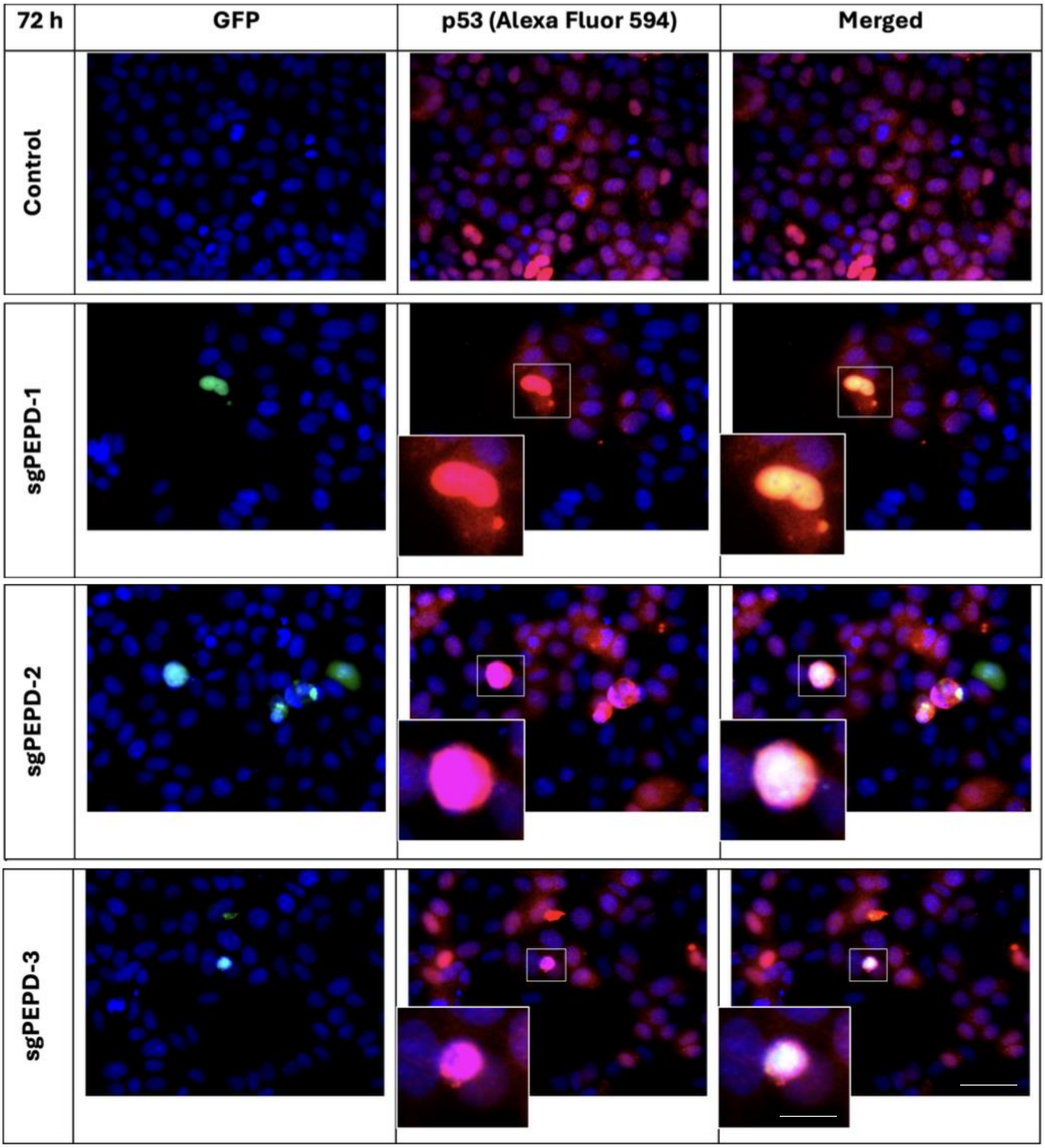
72 h
GFP
p53 (Alexa Fluor 594)
Merged
Control
sgPEPD-1
sgPEPD-2
sgPEPD-3

B

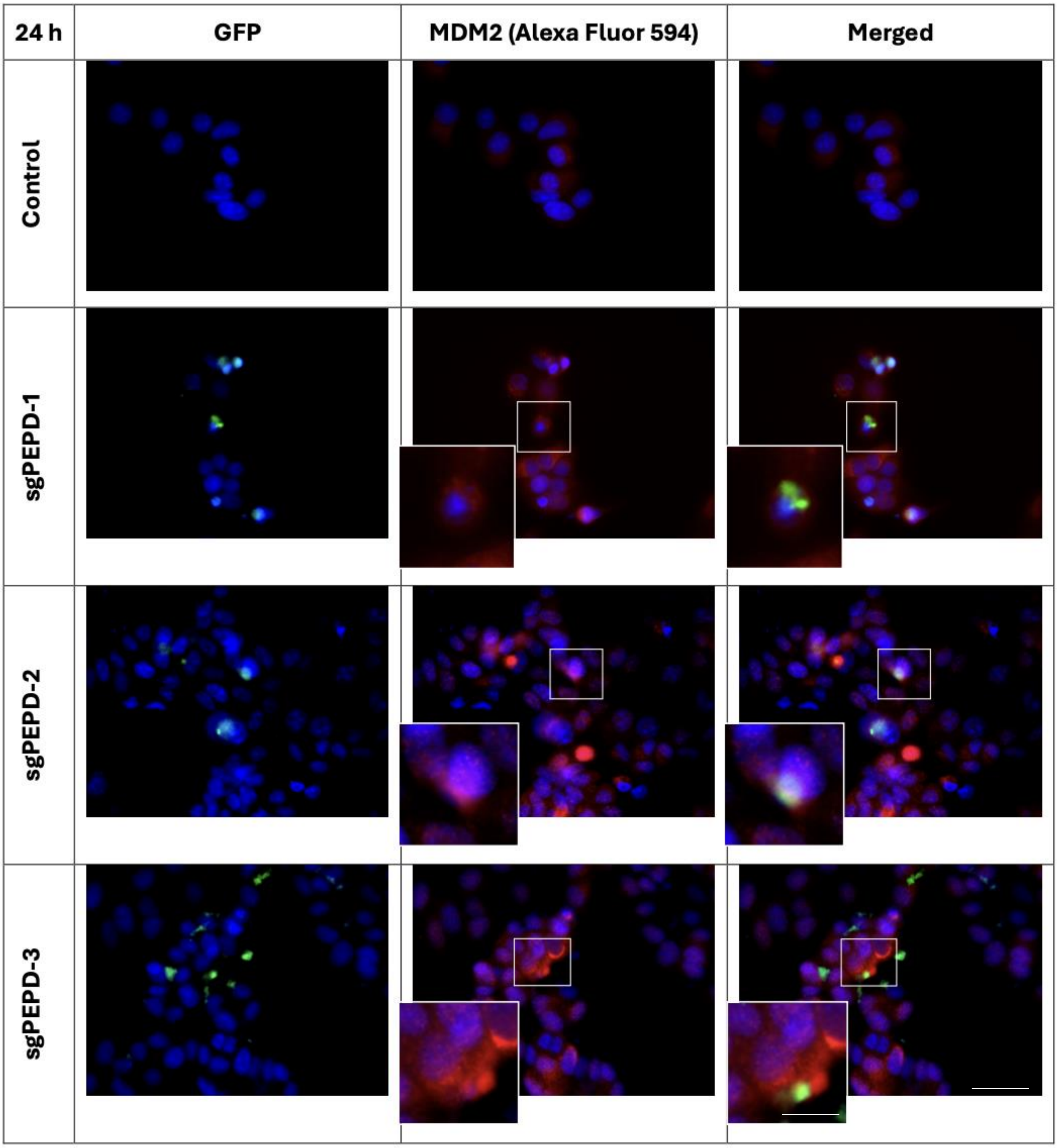
24 h
GFP
MDM2 (Alexa Fluor 594)
Merged
Control
sgPEPD-1
sgPEPD-2
sgPEPD-3

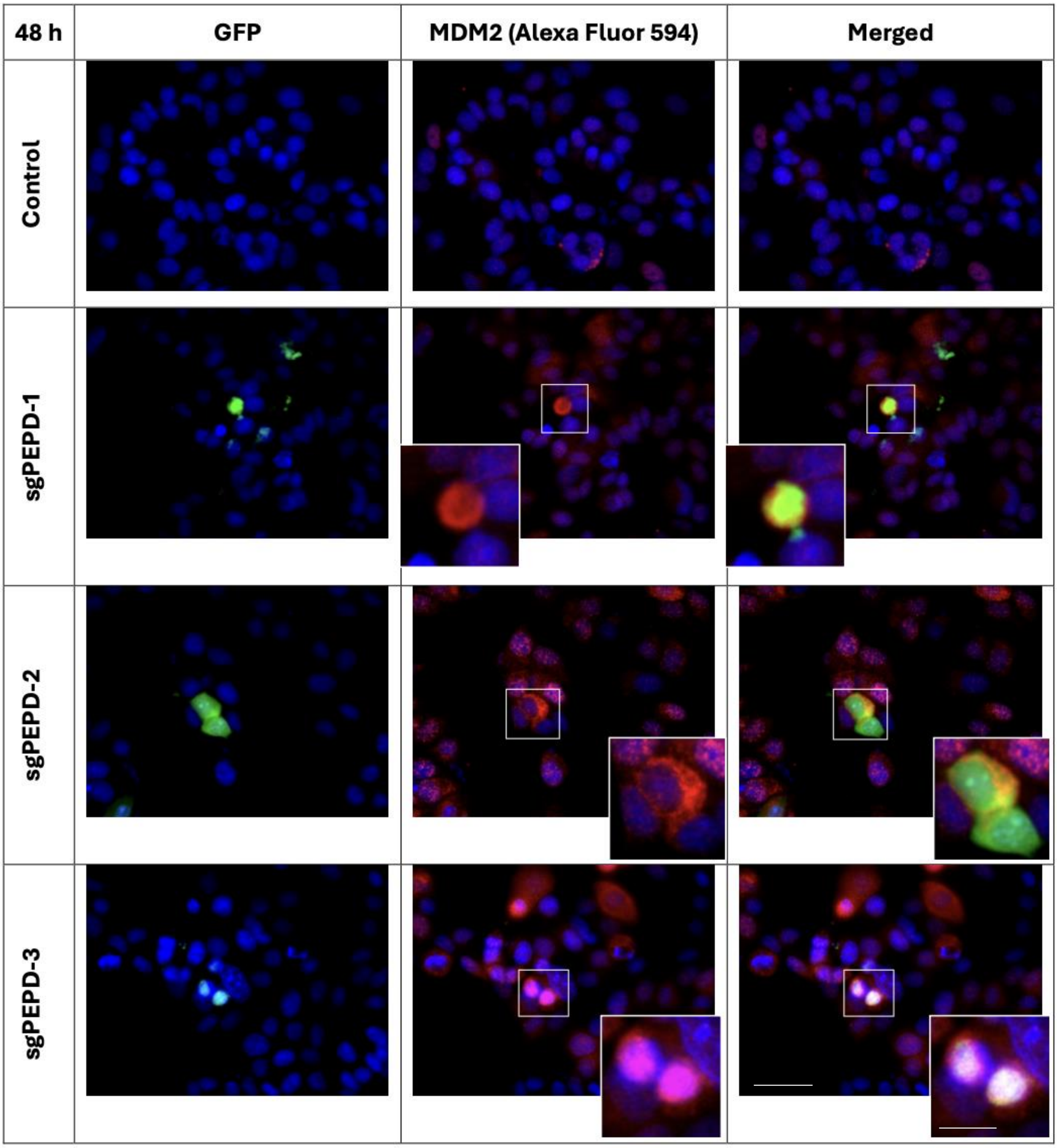
48 h
GFP
MDM2 (Alexa Fluor 594)
Merged
Control
sgPEPD-1
sgPEPD-2
sgPEPD-3

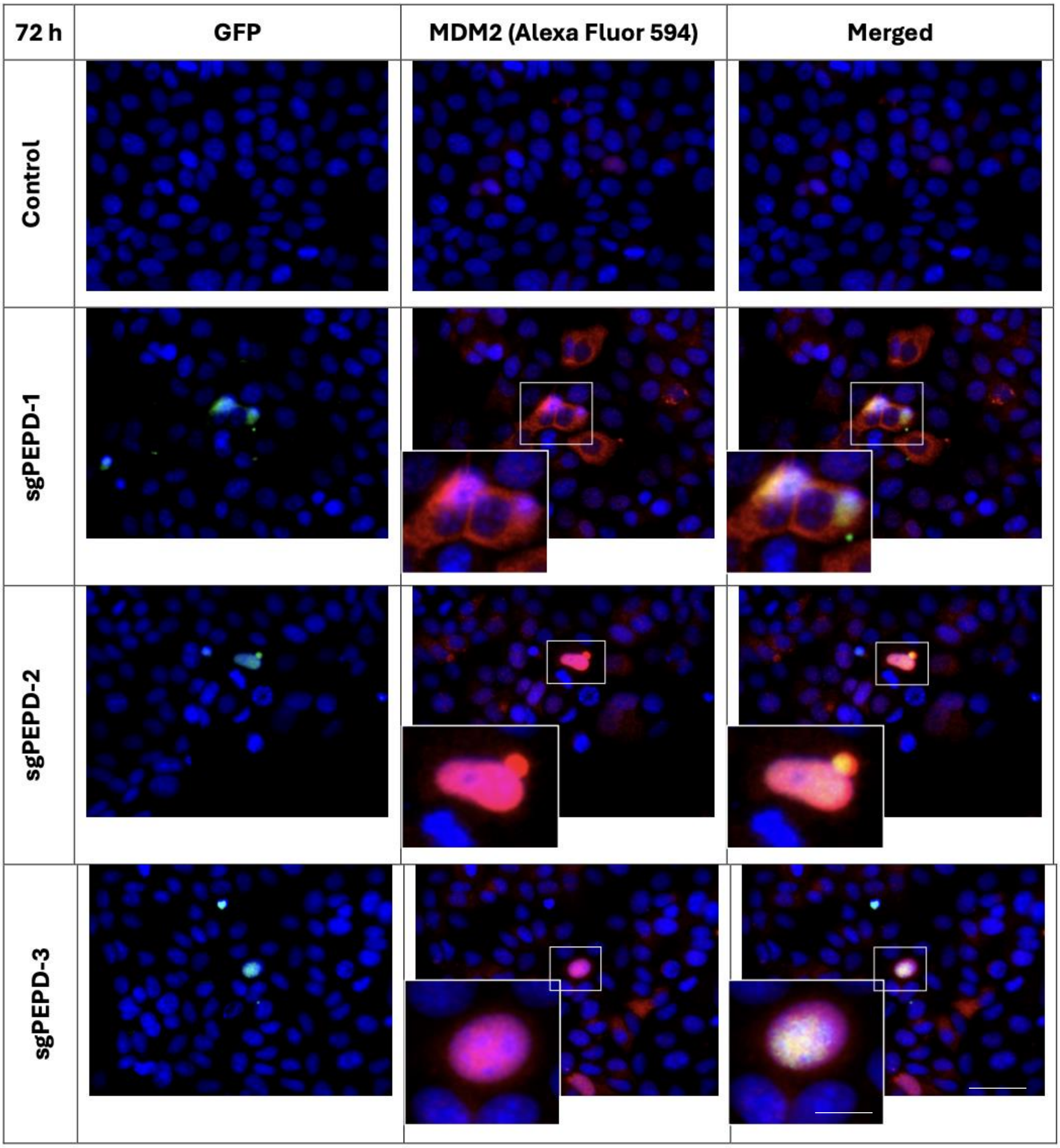

Figure S4. Representative fluorescence microscopy images showing the expression and intracellular localization of p53 in control MCF7[WT] cells and cells transfected with CRISPR/Cas9 constructs targeting the *PEPD* gene (A). GFP fluorescence (green) identifies successfully transfected cells, while p53 expression was visualized using secondary antibodies conjugated with Alexa Fluor 594 (red). Cell nuclei were stained with Hoechst 33342 (blue). Images were acquired 24, 48, and 72 hours after transfection using an Olympus 40x objective. (B) Representative fluorescence microscopy images showing MDM2 immunoreactivity in untreated MCF7 reference cells and cultures transfected with PEPD-targeting CRISPR/Cas9 constructs. GFP fluorescence (green) indicates detectable plasmid expression, MDM2 was

visualized with Alexa Fluor 594 (red), and nuclei were stained with Hoechst 33342 (blue). White boxes indicate the regions shown at higher magnification in the corresponding enlarged insets. Insets were generated by cropping the indicated regions from the respective original images and enlarging them for visualization only; no additional image processing was applied specifically to the inset regions. Scale bars: 50 μm in the main images and 10 μm in the enlarged insets.
