# Supplementary material for: CRISPR/Cas9-Directed PEPD Targeting Is Associated with Reduced PEPD Activity and an Exploratory p53- and Apoptosis-Related Protein Pattern in MCF7 Breast Cancer Cells
Source: Molecules. 2026 Sep 22;31(19):3360. doi: 10.3390/molecules31193360 (PMC13666405; doi:10.3390/molecules31193360)
Supplement: Supplementary file 1 [file molecules-31-03360-s001.zip › Supplementary Figure S5.pdf]

A

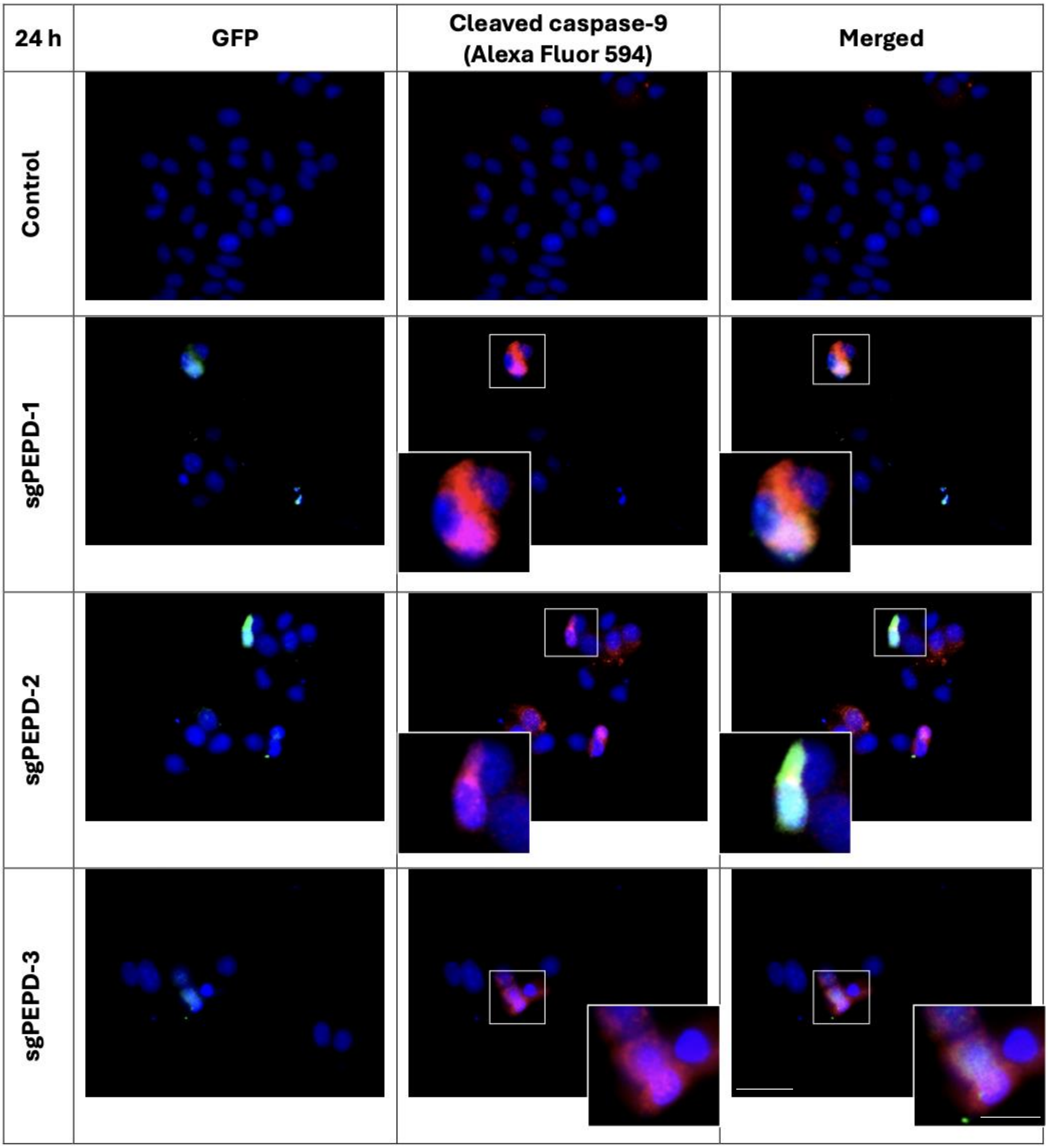
24 h
GFP
Cleaved caspase-9 (Alexa Fluor 594)
Merged
Control
sgPEPD-1
sgPEPD-2
sgPEPD-3

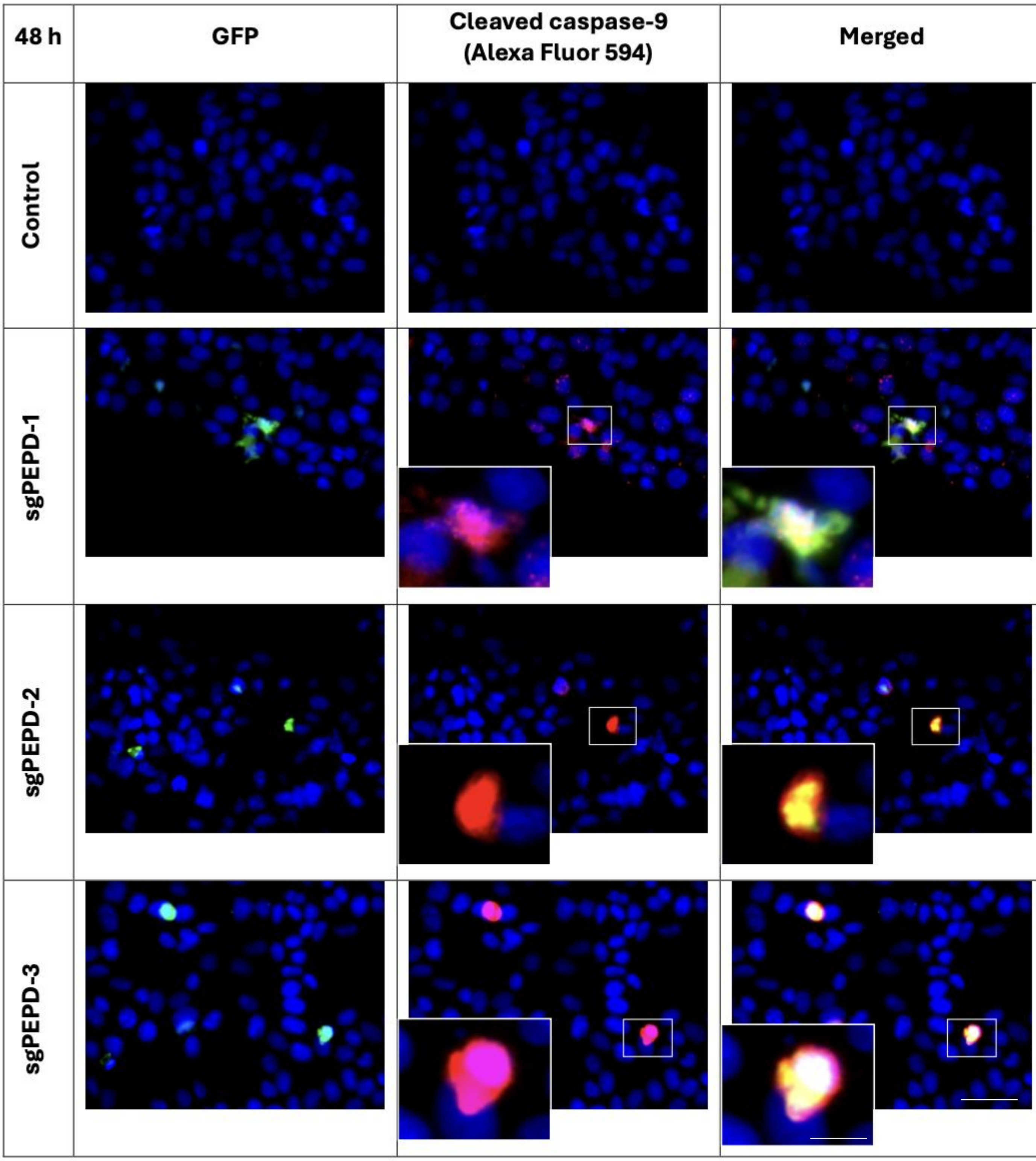
48 h
GFP
Cleaved caspase-9
(Alexa Fluor 594)
Merged
Control
sgPEPD-1
sgPEPD-2
sgPEPD-3

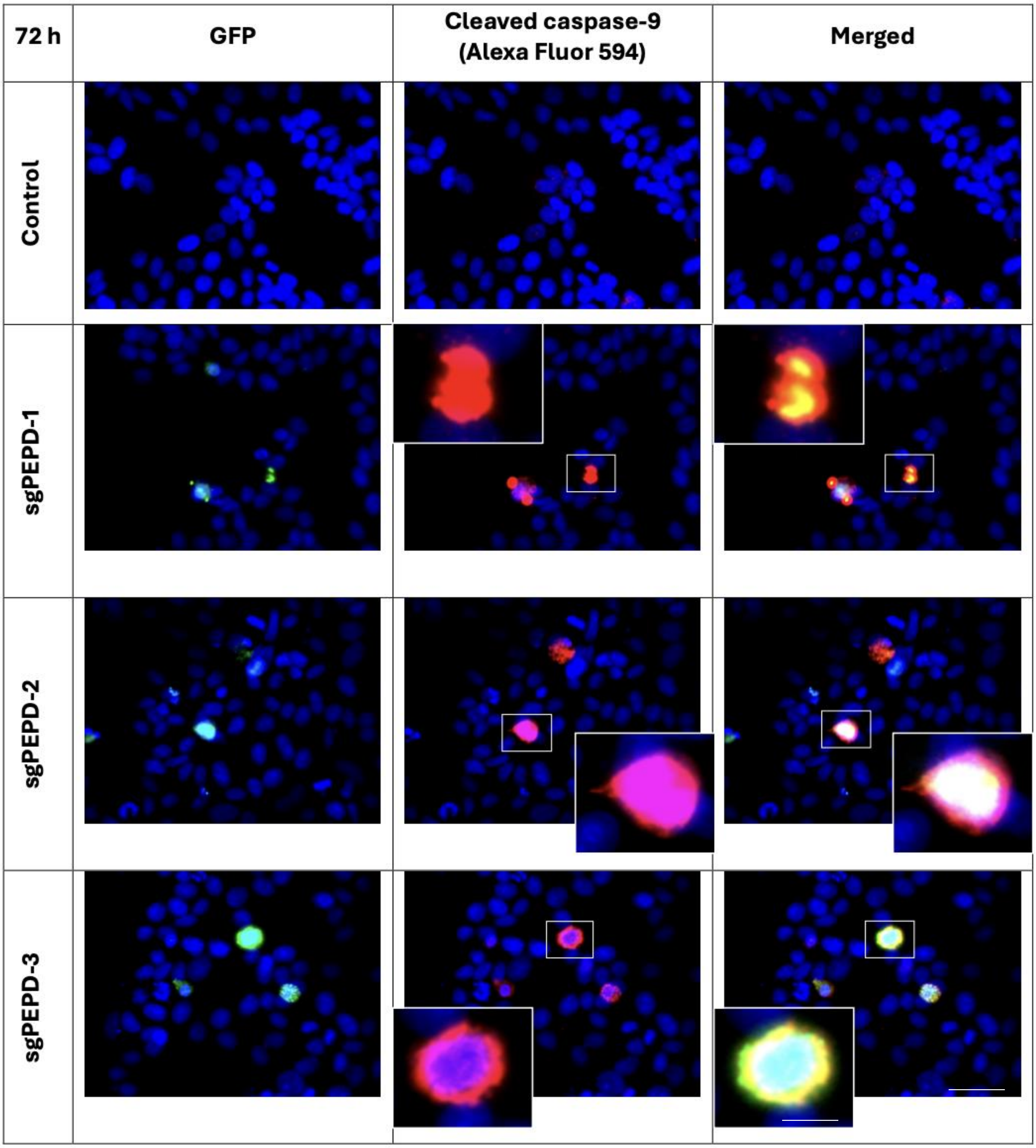
72 h
GFP
Cleaved caspase-9 (Alexa Fluor 594)
Merged
Control
sgPEPD-1
sgPEPD-2
sgPEPD-3

B

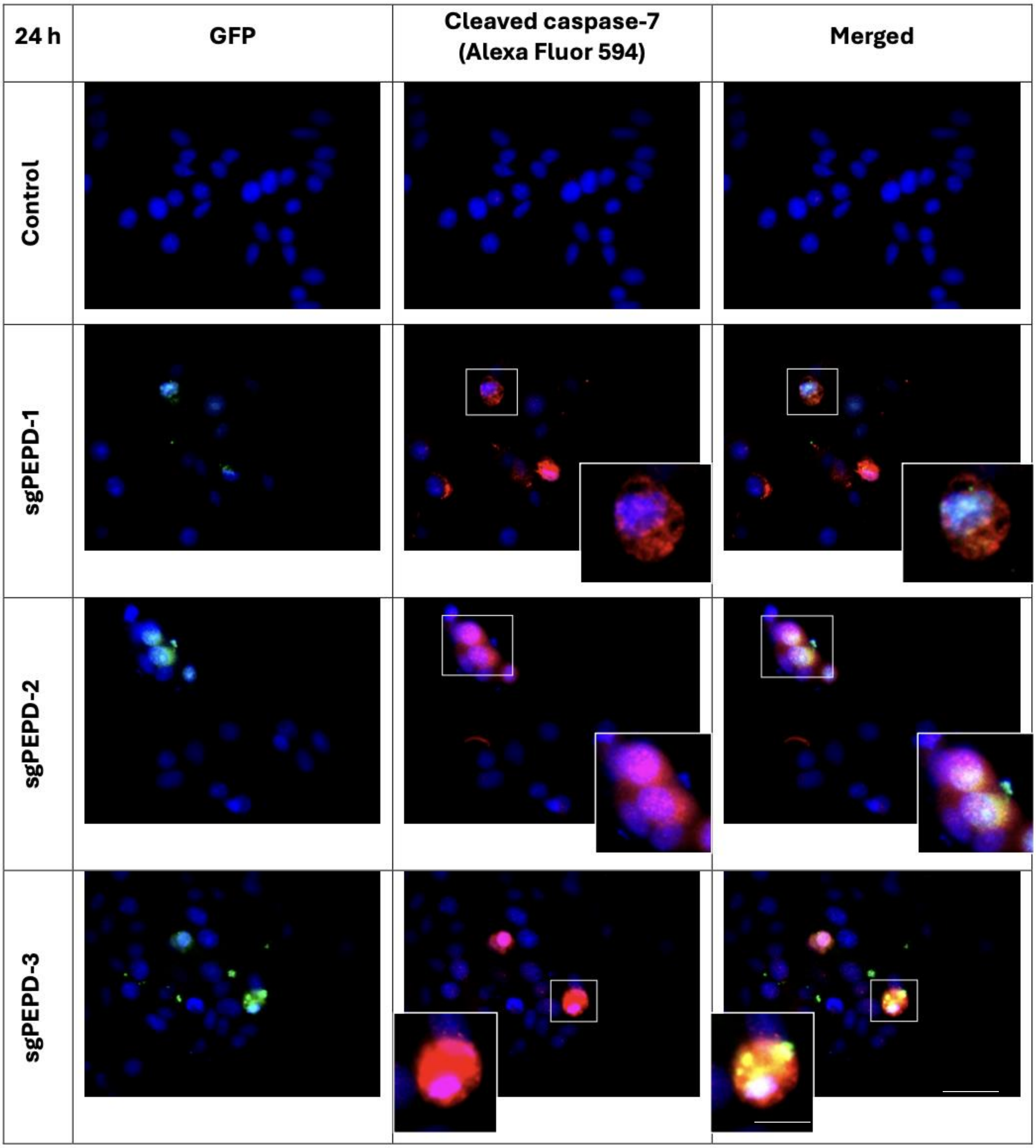
24 h
GFP
Cleaved caspase-7
(Alexa Fluor 594)
Merged
Control
sgPEPD-1
sgPEPD-2
sgPEPD-3

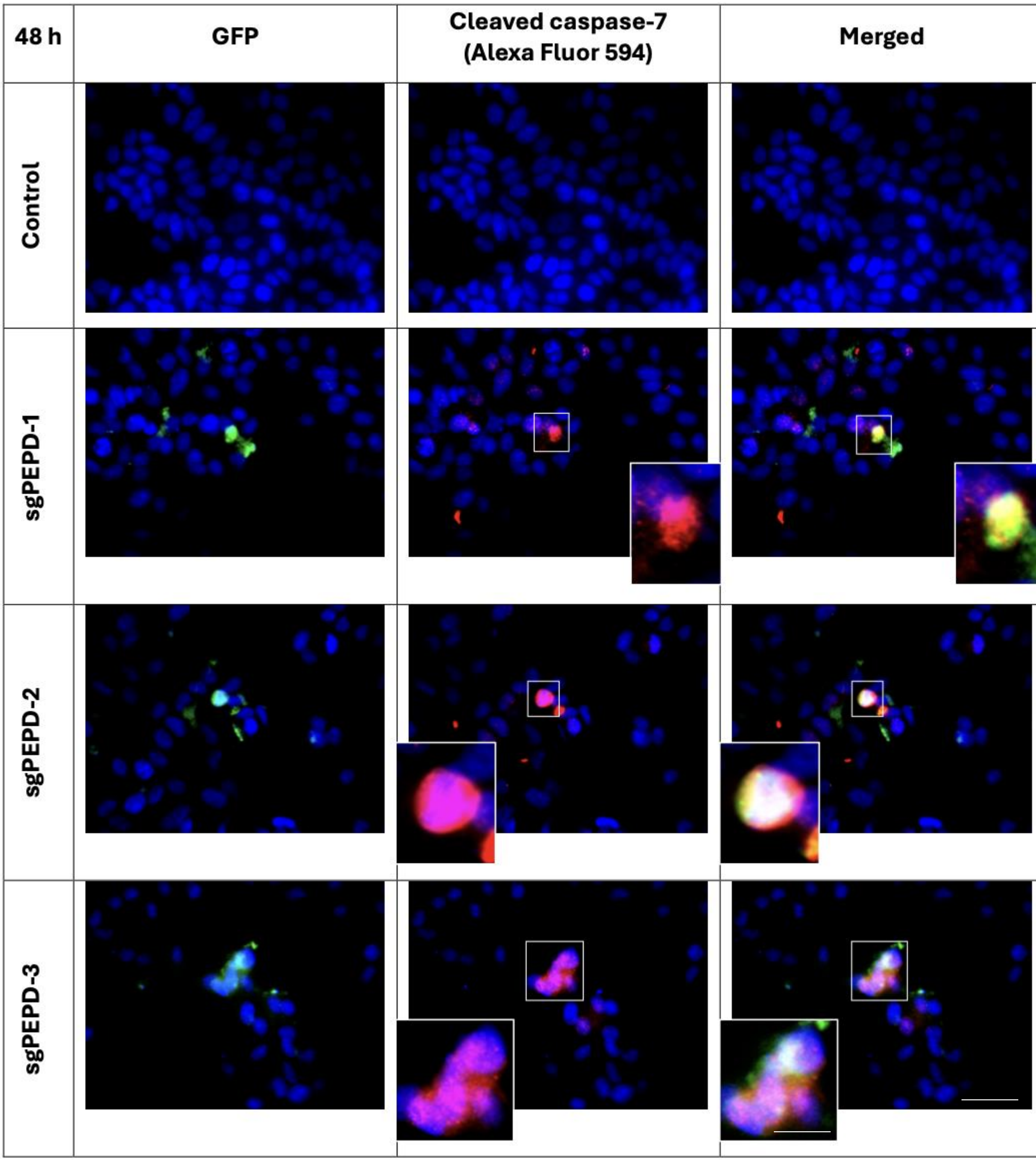
48 h
GFP
Cleaved caspase-7 (Alexa Fluor 594)
Merged
Control
sgPEPD-1
sgPEPD-2
sgPEPD-3

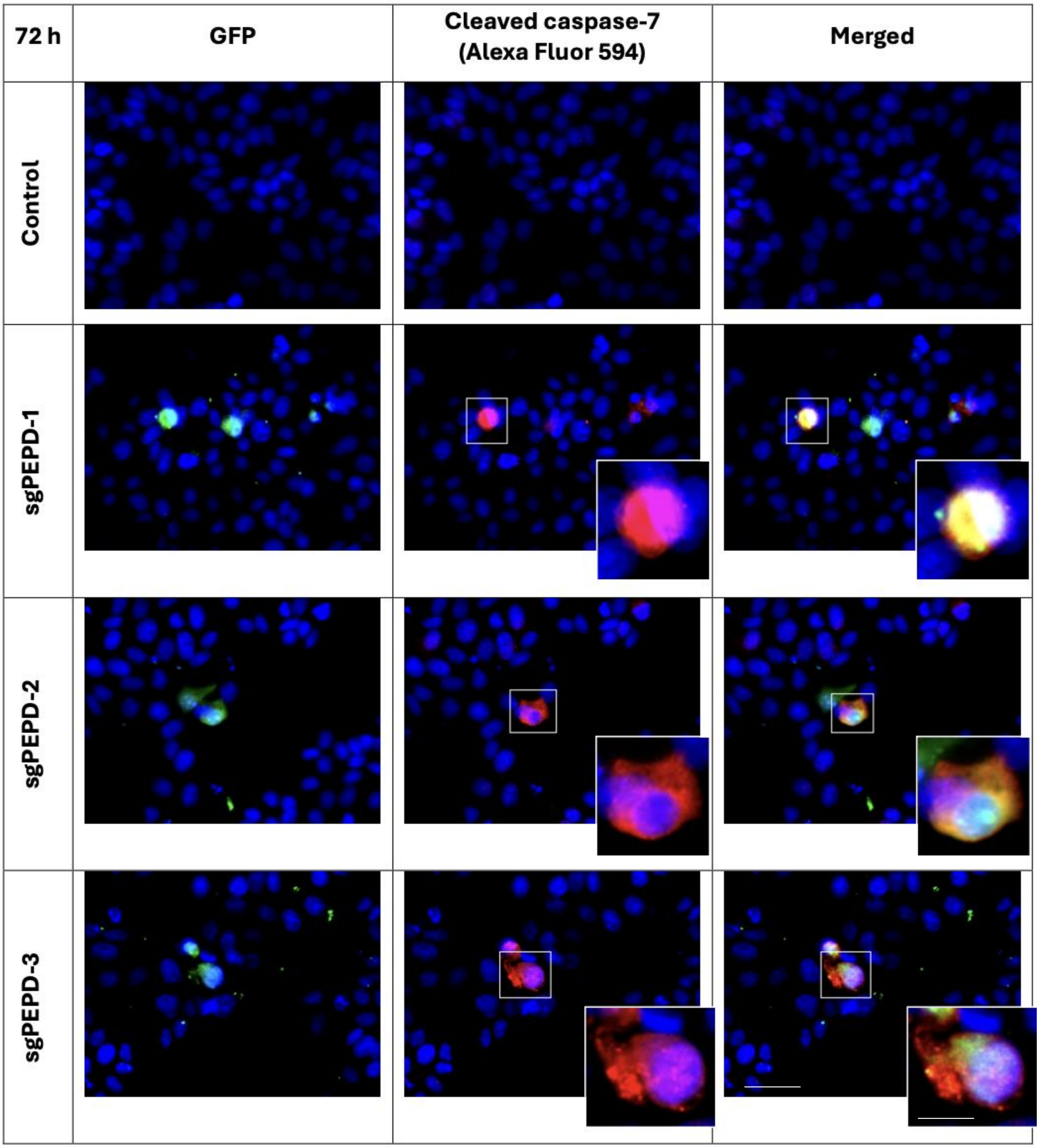
72 h
GFP
Cleaved caspase-7 (Alexa Fluor 594)
Merged
Control
sgPEPD-1
sgPEPD-2
sgPEPD-3

C

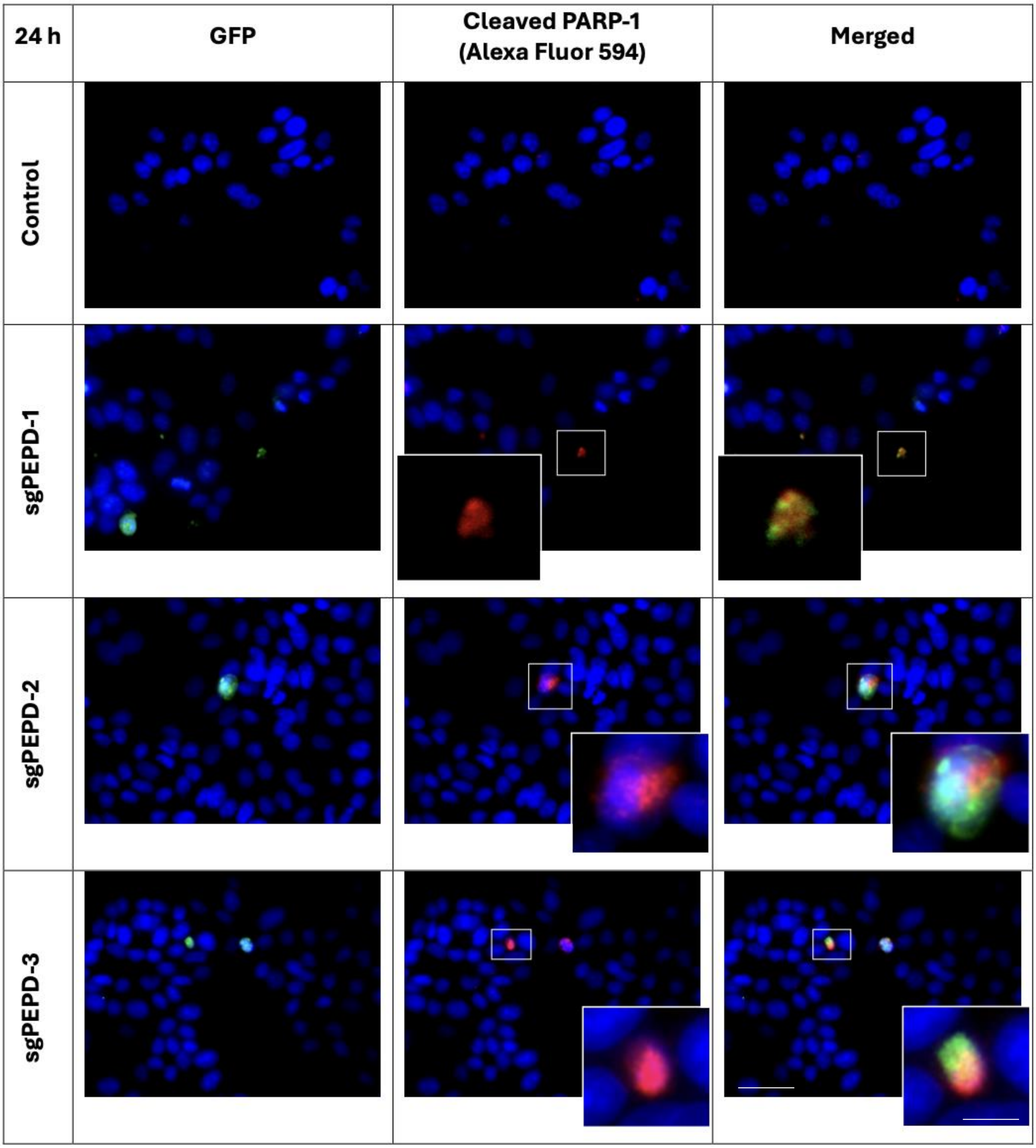
24 h
GFP
Cleaved PARP-1
(Alexa Fluor 594)
Merged
Control
sgPEPD-1
sgPEPD-2
sgPEPD-3

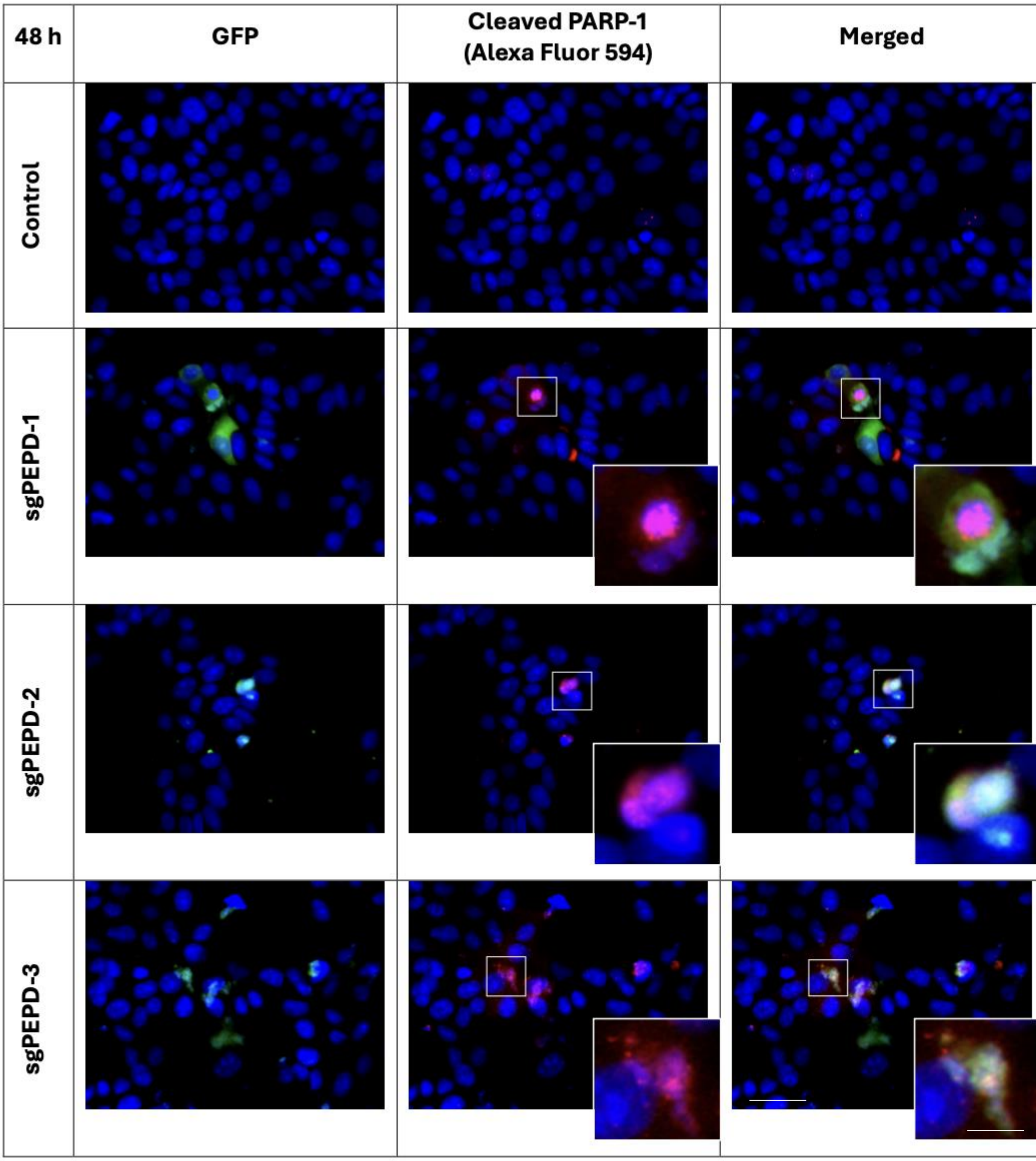
48 h
GFP
Cleaved PARP-1 (Alexa Fluor 594)
Merged
Control
sgPEPD-1
sgPEPD-2
sgPEPD-3

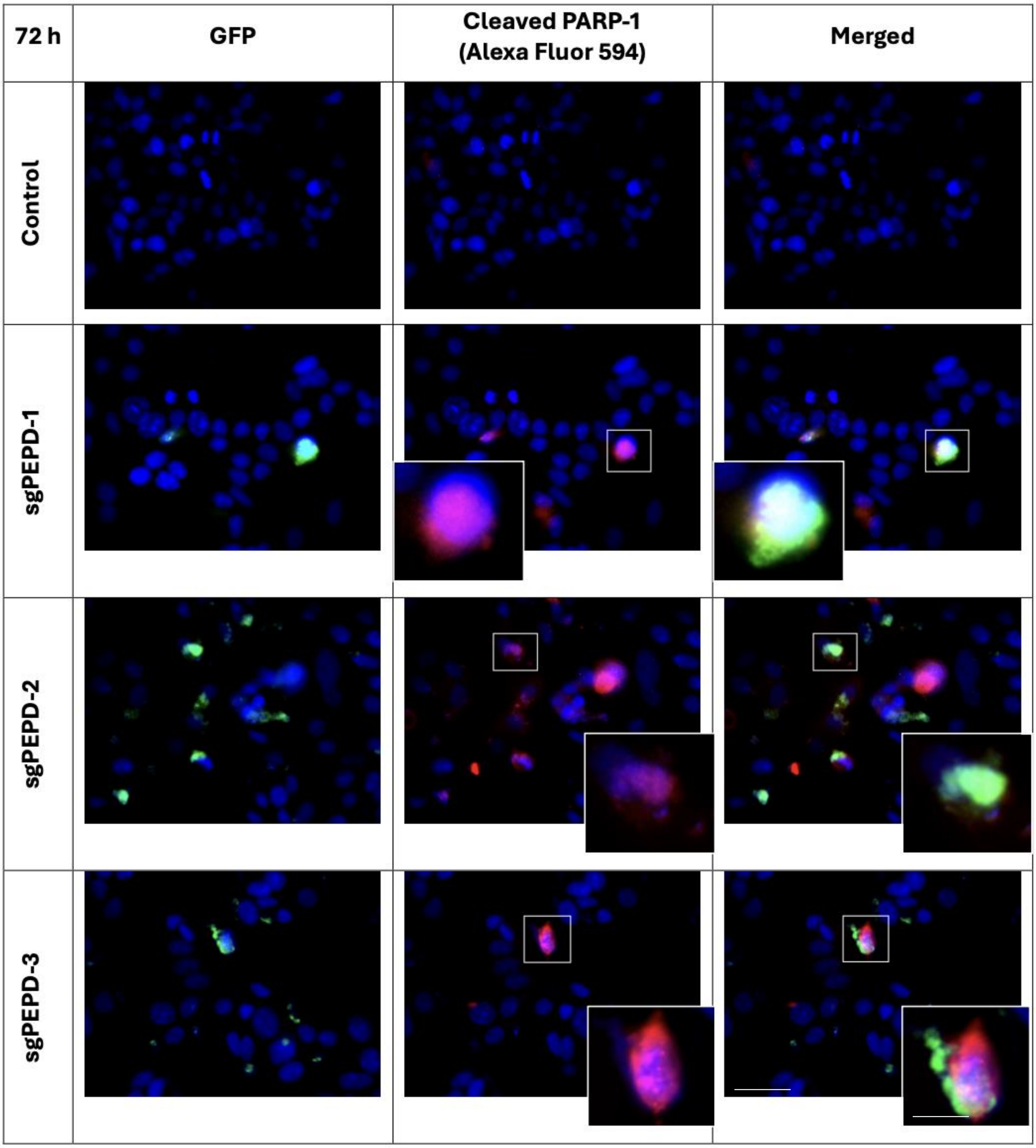

Figure S5. Representative fluorescence microscopy images showing the expression of cleaved caspase-9 (A), cleaved caspase-7(B) and cleaved PARP-1 (C) proteins in MCF7[WT] control cells and cells transfected with CRISPR/Cas9 constructs targeting the *PEPD* gene. GFP fluorescence (green) identifies successfully transfected cells, while cleaved caspase-9 expression was visualized using secondary antibodies conjugated with Alexa Fluor 594 (red). Cell nuclei were stained with Hoechst 33342 (blue). Images were acquired 24, 48, and 72 hours after transfection using an Olympus 40x objective. **(C)** Representative fluorescence microscopy images showing the expression of activated caspase-7 in control MCF7[WT] cells and cells transfected with CRISPR/Cas9 constructs targeting the *PEPD* gene. GFP fluorescence (green)

identifies successfully transfected cells, while the expression of cleaved caspase-7 was visualized using secondary antibodies conjugated with Alexa Fluor 594 (red). Cell nuclei were stained with Hoechst 33342 (blue). Images were acquired 24, 48, and 72 hours after transfection using an Olympus 40x objective. **(D)** Representative fluorescence microscopy images showing the expression of cleaved PARP-1 in control MCF7^{WT} cells and cells transfected with CRISPR/Cas9 constructs targeting the *PEPD* gene. GFP fluorescence (green) identifies successfully transfected cells, while cleaved PARP-1 expression was visualized using secondary antibodies conjugated with Alexa Fluor 594 (red). Cell nuclei were stained with Hoechst 33342 (blue). The images were taken 24, 48, and 72 hours after transfection using an Olympus 40x objective. White boxes indicate the regions shown at higher magnification in the corresponding enlarged insets. Insets were generated by cropping the indicated regions from the respective original images and enlarging them for visualization only; no additional image processing was applied specifically to the inset regions. Scale bars: 50 μm in the main images and 10 μm in the enlarged insets.
