# Supplementary material for: CRISPR/Cas9-Directed PEPD Targeting Is Associated with Reduced PEPD Activity and an Exploratory p53- and Apoptosis-Related Protein Pattern in MCF7 Breast Cancer Cells
Source: Molecules. 2026 Sep 22;31(19):3360. doi: 10.3390/molecules31193360 (PMC13666405; doi:10.3390/molecules31193360)
Supplement: Supplementary file 1 [file molecules-31-03360-s001.zip › Supplementary Figure S6.pdf]

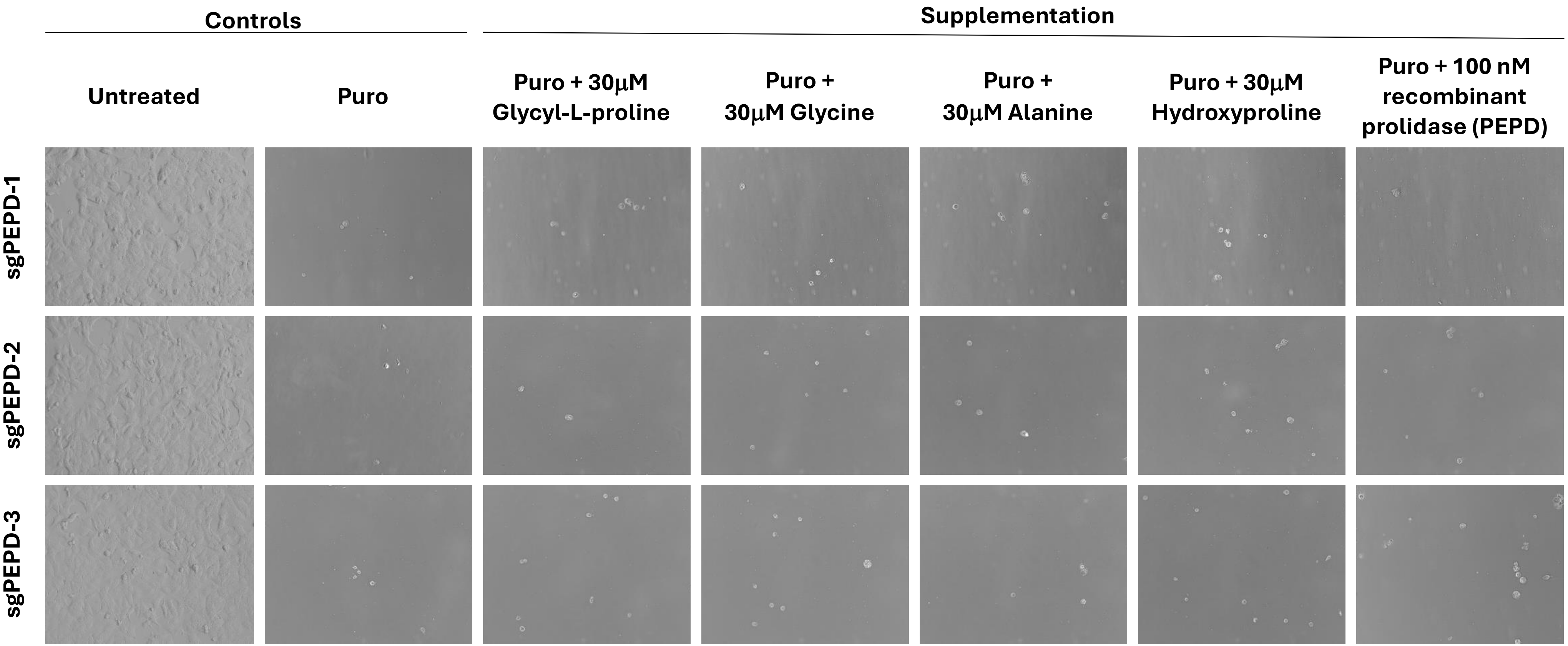

Supplementary Figure S6. Glycyl-L-proline, amino acid supplementation, and recombinant PEPD do not permit maintenance of puromycin-resistant MCF7 populations after CRISPR/Cas9-mediated PEPD targeting.

Representative brightfield images of MCF7 cells transfected with three independent CRISPR/Cas9-Puro constructs targeting the PEPD gene (sgPEPD-1, sgPEPD-2, and sgPEPD-3) and subjected to prolonged puromycin selection. Cells were cultured in the absence of additional supplementation (Puro) or in medium supplemented with 30 μM glycyl-L-proline (Gly-Pro), 30 μM glycine, 30 μM alanine, 30 μM hydroxyproline, or 100 nM recombinant PEPD. Untreated MCF7 cells were included as a reference condition. Puromycin was used at a concentration of 1.5 μg/mL. Images were acquired 10 days after transfection, following 7 days of puromycin selection and a subsequent 3-day observation period. Under the experimental conditions tested, none of the supplemented compounds permitted maintenance of an adherent puromycin-resistant population for any of the three PEPD-targeting sgRNA constructs. The images provide qualitative documentation of the persistence of adherent selected cells and were not used as a quantitative measure of cell viability, apoptosis, or cell death.
